# Supplementary material for: Health system assessment for access to care after injury in low- or middle-income countries: A mixed methods study from Northern Malawi
Source: PLoS Med. 2024 Jan 22;21(1):e1004344. doi: 10.1371/journal.pmed.1004344 (PMC10843098; doi:10.1371/journal.pmed.1004344)
Supplement: S1 GRAMMS Checklist — (DOCX) [file pmed.1004344.s001.docx]

Supplementary Material 2 – Good Reporting of A Mixed Methods Study (GRAMMS) [1]

| (1) Describe the justification for using a mixed methods approach to the research question | Introduction paragraph 3. |
| --- | --- |
| (2) Describe the design in terms of the purpose, priority and sequence of methods | Methods section, study design paragraphs. |
| (3) Describe each method in terms of sampling, data collection and analysis | Table 1 and S1 Unpublished Methods Supplementary Material. |
| (4) Describe where integration has occurred, how it has occurred and who has participated in it | Methods section, mixed methods analysis paragraphs. |
| (5) Describe any limitation of one method associated with the present of the other method | Limitations paragraph within discussion. |
| (6) Describe any insights gained from mixing or integrating methods | Discussion section as a whole |

Reference:

1. O'cathain A, Murphy E, Nicholl J. The Quality of Mixed Methods Studies in Health Services Research. *Journal of Health Services Research & Policy*. 2008;13:92-8.
