# Supplementary material for: Health system assessment for access to care after injury in low- or middle-income countries: A mixed methods study from Northern Malawi
Source: PLoS Med. 2024 Jan 22;21(1):e1004344. doi: 10.1371/journal.pmed.1004344 (PMC10843098; doi:10.1371/journal.pmed.1004344)
Supplement: S1 Text — (DOCX) [file pmed.1004344.s002.docx]

**S1 Unpublished Methods Supplementary Material**

## Household Survey

### Aim

To describe the non-fatal injury burden and subsequent health system utilization within the Karonga Health and Demographic Surveillance Site (HDSS) community.

### Objectives

1. Describe the burden of non-fatal injury within the HDSS.
2. Describe barriers and delays to care experienced following injury.
3. Describe household estimates of time taken to access health facilities in an emergency.

### Survey development

The survey was adapted from the World Health Organisation’s (WHO) Guidelines for conducting community surveys on injuries and violence [1]. Additional questions were included to capture health-seeking behaviour, experience of, and reasons for delays to seeking, reaching and receiving healthcare and perceived healthcare quality. The survey was translated into the vernacular language of Chitumbuka by trained native speakers. It was then back-translated to confirm accuracy of meaning.

Ten professional research fieldworkers, native Chitumbuka speakers, were trained over a week in administering the survey. The survey was piloted within the local community on a sample of households not included in the formal study, known to include individuals with recent experience of an injury. Following piloting, minor adjustments were made to question layout and Chitumbuka phrasing to improve comprehension and clarity.

### Sample size

To determine sample size, we anticipated a non-fatal injury annual incidence of approximately 5% and 0.5% for minor and major injuries respectively, which have been found in other sub-Saharan Africa settings [2-4]. To draw meaningful conclusions about healthcare utilisation and experience amongst the population we judged 100 episodes of healthcare utilization for injury to be a reasonable minimum requirement. This is comparable to the sample size used in other studies of health system experience [5-8]. we assumed a conservative estimate of healthcare utilisation following injury of 20% and a non-fatal injury incidence of 5%. Therefore, a survey covering 10,000 people was anticipated to identify 500 injured people of whom 100 would have sought care. This sample size is comparable to that used in other household surveys evaluating injury-related morbidity and healthcare utilization [8]. Since the HDSS households contain five individuals on average, 2,000 households were required. Experienced HDSS staff estimated 10% of local households might refuse or be unavailable to take part. Therefore 2,200 households were randomly selected for this study, approximately one-fifth of the HDSS.

### Identification of participants

Any household member present aged over 18 was eligible to act as a proxy informant to complete the survey on behalf of the household. Proxy responses have been used effectively in other studies when a household member who had experienced an injury is absent or unable to complete the survey or is a child [3]. If no such household member was present, fieldworkers enquired from neighbours if they were somewhere close by, in which case they visited them there. Otherwise, fieldworkers undertook one further visit while working in the same household cluster. If there was still no adult household member present, the household was marked as missing. If more than one willing adult over 18 was present during the visit, the one most knowledgeable about recent injuries within the household was selected.

### Data collection

The survey was administered using REDCap [9] data collection mobile application using password-protected tablets. Data was uploaded to a central, secure server held at the Malawi Epidemiology and Intervention Research Unit’s (MEIRU) Chilumba research site every evening after fieldwork and the tablet reset ready for reuse.

The survey was divided into two sections. The first seven questions were asked of all households. The second section was only applicable if the household reported at least one non-fatal injury in the preceding 12 months. In the first section of the survey the informant was asked the following: which facility they would prefer to go to if they were to suffer an injury, with allowed categories of named local facilities and other specified; to estimate how long it would take to get to their preferred facility in an emergency in minutes; whether this was the closest facility, and if not, which facility was closest (named facility or other specified), and how long it would take to get to the closest facility in an emergency in minutes; how long in minutes it would take to get to both Karonga District Hospital and Mzuzu Central Hospital; and whether or not there had been either a fatal or non-fatal injury in the preceding 12 months, and if so, how many. Injury was defined as physical damage following any external force. These questions were asking the participant to estimate the typical time to reach the location in an emergency. Exactly how participants would choose and arrange to travel would be for them to decide and estimate and was not specifically explored.

For households without any non-fatal injuries in the preceding 12 months, the survey ended. For households with at least one non-fatal injury reported, section two of the survey questionnaire was completed for each injured person. Fatal injury insights were obtained through verbal autopsy analysis described later. A non-fatal injury minimum severity was defined as preventing the injured person from performing their usual activities for at least one day or seeking healthcare. If an individual had suffered more than one injury in the preceding 12 months, only the most severe injury, defined as that with the largest number of days unable to perform usual activities, was recorded.

In the second section of the survey, the informant was asked; how long ago the injury occurred (in months), sex, the age of the injured person in years, whether or not the injured person has returned to full activity (exclusive categories of yes fully, yes but only partially and no; partial recovery meant returning to activities with an ongoing limitation not previously present), and how long in total they were not able to perform normal activities in days.

The informants were asked in what way the injured person was physically impaired (able to select multiple categories from unable to use their hand or arm, difficulty in using their hand or arm, unable to walk, difficulty in walking, loss of hearing, loss of vision, weakness or shortness of breath, less able to remember things, unable to eat without assistance, and any other specified with free text). Mechanism of injury was recorded (exclusive categories of road traffic accident, fall, struck/hit by person or object, stab, gunshot, fire, flames or heat, drowning or near-drowning, poisoning, animal bite, electric shock, other specified with free text or unknown). The physical injuries sustained were recorded (able to select multiple categories from a fracture, a sprain or strain, joint dislocation, cut, bite or other open wound, burn, poisoning, concussion or head injury, internal injury or internal organ injury to their chest, internal injury or internal organ injury to their abdomen or pelvis, or other specified with free text). Informants were asked whether the injured person sought medical attention or treatment outside of the household. If the answer was yes, a series of questions about health system utilisation were asked. If the answer was no, a series of questions about health-seeking choices was asked.

For those injured persons who had sought medical treatment, the informants were asked where they first sought care (named facility or other specified with free text), whether they attended a second facility and if yes where (named facility or other specified with free text). How long after injury the person decided to seek care, how long it took to reach the first care facility after deciding to seek, and how long it took before starting to receive treatment once at the first facility attended (exclusive categories of <1 hour, 1-2 hours, 2-4 hours, 4-6 hours, 6-12 hours, 12-24 hours, >24 hours) were recorded. The delay in receiving treatment was the time between arriving at a facility and actually receiving something considered treatment from a healthcare worker, such as medications, intravenous (IV) fluids or wound care. Informants were asked whether the injured person stayed overnight in a health facility and if yes for how many days; and how satisfied the treated person was that the care received was good quality was recorded (exclusive categories of very satisfied, satisfied, neither satisfied nor dissatisfied, dissatisfied or very dissatisfied).

For those injured persons who had not sought medical care the reasons why were asked (able to select multiple categories from “The injury was not serious enough to need medical care”, “The person or family had other priorities or responsibilities”, “It was too difficult to get transport to health facility”, “The family member responsible for decisions about seeking care did not want the injured person to seek care”, “The financial cost of seeking care was too much”, “The health facility was too far away”, “The injured person prefers to see traditional healers for health problems”, “The health facility would not provide effective treatment for this problem”, “The injured person did not believe that is was right to seek care following an injury”, “The health facility would not treat the injured person with respect”, “The injured person did not know healthcare was available”, “People fear the consequences of helping an injured person (e.g. being accused of causing the injury)”, “The health facility would not communicate well with the injured person and family” and other specified with free text). From the same reasons, the most important was also identified by participants.

Participants were asked whether or not the injured person lost their job as a result of the injury (mutually exclusive categories “yes”, “no, kept the job” and “no, did not have a job to lose”) and whether anyone else in the household lost days away from normal activities including work or school to take care of them.

### Outcome variables

The primary outcome for this analysis is the incidence of at least one non-fatal injury stratified by major and minor. As is common in household studies on nonfatal injuries,[3] injuries were categorised as major or minor based on the number of days of disability. Injuries with an associated disability of between 1 and 29 days are minor, those with 30 days or more are major. The number of occupants for households surveyed (incidence denominator) was taken from available HDSS surveillance data. Secondary outcomes are time delays to seeking, reaching and receiving care, barriers preventing seeking care and patterns of facility preference and use.

Delays to care were classified into those receiving care in less than the “Golden Hour” (seeking, reaching and receive care all took <1 hour), less than the Lancet Commission on Global Surgery (LCoGS) 2-hour target but more than 1 hour (two of either seeking, reaching or receiving care took < 1hour and the other took 1-2 hours), and longer than 2 hours (any other combination of delay times). Free text responses for other specified answers were reviewed by one author (JW). Where suitable these were assigned to an existing category or a new category was assigned. Facilities named were categorised as “government primary”, “government secondary”, “government tertiary”, “faith-based primary”, “faith-based secondary”, “military primary”, “private primary” and “unknown”. All the faith-based facilities were part of the Christian Health Association of Malawi (CHAM). Facilities were further categorised into facility type as primary or referral (including secondary and tertiary care). Whether or not an injured person lost their job was divided into those who had a job to lose and those that didn’t. Mechanism of injury and physical injuries sustained are reported as collected, described above.

### Analysis

Counts, percentages, median and interquartile ranges, describe the results. For those sustaining an injury, we performed univariable analysis using chi-squared (or fisher’s exact if cell counts <5) to test for associations between sustaining a major injury rather than minor injury and whether the patient lost a job they had, required other household members to take time to care for them or not, sought care or not, stayed overnight in a facility or not, attended a second place of care or not, and first facility type visited. For those sustaining an injury, we performed logistic regression multivariable analysis to ascertain factors associated with a major rather than minor injury. Variables included were age, sex, mechanism of injury (categories with count <5 were grouped with other) and physical injuries sustained. Odds ratios, 95% confidence intervals (CI) and p values were calculated.

## Verbal Autopsy

### Aim

To describe the health system utilisation and potential avoidability associated with fatal injury within the Karonga HDSS population, and to identify and categorise any barriers driving delays to care within a “Three Delays” framework.

### Objectives

1. Describe the burden of fatal injury within the HDSS.
2. Describe the proportion of fatal injuries potentially avoidable.
3. Describe barriers and delays to care experienced following avoidable fatal injury.

### Data Collection

Verbal Autopsies (VA) are collected routinely as part of population surveillance within the Karonga HDSS [10]. All members of households located within the geographic area are eligible for inclusion in the demographic surveillance, with only 1% typically declining to take part usually for religious or spiritual reasons [10]. As described earlier, key informants for each cluster are asked to report deaths in addition to other household changes at monthly reporting sessions. All deaths are followed by VA using a semi-structured questionnaire following a suitable period of mourning, typically a few weeks [11]. A clinically trained interviewer then visits the household and completes a semi-structured VA form if the family consent. The VA survey is based on the 2003 INDEPTH and WHO tools [12]. Since the start of the Karonga HDSS, the VA tool used has been modified to reflect changes in the WHO standardised questionnaire. However, the questions regarding deaths from external causes remain unchanged [13]. Relatives who were present at, or leading up to, death are asked open-ended questions to create a narrative about the circumstances of death. This is accompanied by closed symptom-specific questions. Two clinicians independently review the questionnaire to assign a cause of death with a third acting as a tie-breaker in case of disagreement [14].

All VAs available within the Karonga HDSS database from inception in 2002 until September 2019 were searched for external causes of death. External causes of death classified as either underlying, contributory, or direct, within the HDSS database were extracted. All VAs are assigned an underlying cause (initiating the train of morbid events) whilst direct and contributory causes may not always apply. We included all underlying, direct and contributory external causes to review all VAs in which injury played a role. Within the Karonga HDSS external causes are classified as unspecifiable, transport, fall, drowning/submersion, exposure to smoke/fire/flames, poisoning/exposure to noxious substance, use of weapon, contact with venomous animals and plants, hanging, choking, other specific unlisted. Free text narratives were reviewed for each. Narratives were only available as photographs of a paper form. Therefore, it was not possible to key-word search the database for any other deaths that may also be related to trauma otherwise classified. Ten circumstances of mortality (CoM) questions to assess household, community and health system determinants of death were included where available. Some had only been collected as part of verbal autopsy sometime after the start of the HDSS [15]. They include questions on recognition of severity, mobilising assets to seek care, access to care and quality of care. They were originally constructed to align with the Three Delays and are listed in S1 Text Table 1. We reviewed all the external cause cases. Those with narratives missing or not clearly describing an external cause of death were excluded. External cause deaths due to poisoning were excluded. The remaining cases were considered traumatic and assessed for avoidability.

### Outcome variables

The primary outcome for this study was the burden of fatal trauma (frequency, age, sex and mechanisms) and the proportion of fatal trauma deaths that were potentially avoidable. Secondary outcomes were the Delay stage and corresponding barriers associated with avoidable deaths and the health system utilisation for fatal injuries within the health system.

The method for evaluating avoidability from VA data has been described before [6]. Each free text narrative was reviewed for descriptions of geographical location of external injury or death, acuity or chronicity of external injury, signs and symptoms after external injury, ability to seek care, factors facilitating or inhibiting access to care, the provision of medical care, results of care delivered, and understanding of the effects of care on the patient’s outcome. Factors indicating potentially avoidable deaths include being alive at the scene of injury, being able to seek help, and low severity injuries affecting a single body system [6]. Deaths were categorised by avoidability and if deemed potentially avoidable, which delay or delays were implicated. Barriers derived from the Delphi study [16] were noted if evidenced, as was any novel barrier believed to be evidenced as driving delays to care. Where multiple delays contributed to avoidable mortality, that found to be the most important in leading to death was considered the primary contributing delay. The categorisations of avoidability and the delays and barriers implicated were performed first in a subsample of 10% of trauma cases. These were then validated by external review with a trauma care provider and researcher with previous low- and middle-income country (LMIC) VA analysis experience to assess avoidability of trauma deaths. Discrepancies were discussed until consensus was achieved to improve the classification of subsequent data. Once agreement was reached on avoidable deaths, delays and barrier classification, we analysed the remaining cases. Whether or not CoM questions helped form the judgement of avoidability or barrier assignment was recorded.

The mechanism of injuries were recategorized using the external cause classification and free text narratives to match those used in the household survey (fall, struck/hit by person or object, road traffic accident, animal bite, fire/flames or heat, stab, drowning or near-drowning, other and unknown). Facilities attended were classified as in the household survey as “government primary”, “government secondary”, “government tertiary”, “faith-based primary”, “faith-based secondary”, “military primary”, “private primary” and “unknown”. They were also summarised as primary, secondary, tertiary and quaternary. Whether or not an injury clearly occurred outside the Karonga HDSS health system was recorded. This excluded those fatally injured who did not access the health system facilities and geographically remote from the Karonga HDSS location (for example deaths occurring when visiting another region).

S1 Text Table 1 Verbal Autopsy Circumstances of Mortality (CoM) questions.

Legend – list of the questions included as circumstances of mortality within the verbal autopsy tool.

| In the final days, were there any doubts about whether the illness was sufficiently serious to need medical care at a health facility? |
| --- |
| In the final days, was traditional medicine used as a major part of the treatment? |
| In the final days, did anyone use a telephone or cell phone to call for help? |
| Was motorised transport (car, truck, tractor, motorcycle, scooter or ambulance) used to get to the hospital or health facility? |
| Does it take more than 2 hours to get to the nearest hospital or health facility offering 24-hour service from the deceased's household? |
| Over the course of illness, did the total costs of care and treatment prohibit other household payments? |
| In the final days, did the deceased travel to a hospital or health facility offering a 24-hour service? |
| Were there any problems (delays, paperwork, queues, no staff etc.) during admission to the hospital or health facility? |
| Were there any problems with the way the deceased was treated (medical treatment, procedures, inter-personal attitudes, respect, dignity) in the hospital or health facility? |
| Were there any problems getting medications, or diagnostic tests in the hospital or health facility? |

### Analysis

Avoidable deaths and the delays and barriers evidenced are described as counts and percentages. For those who accessed a care facility, including multiple facilities, the order and choice of facilities was described.

## Community Focus Group Discussions and Photovoice

### Aim

To establish community perspectives on factors acting as barriers or facilitators to care following injury within a Three Delays conceptual framework through Focus Group Discussion and photovoice methods.

### Data Collection

#### Participant selection

Three community Focus Group Discussions (FGDs) were undertaken. Eight adults (>18 years) were purposively selected for each. The first consisted of members of the general public who had recently (within the past 12 months) sustained an injury with a minimum severity of either being unable to perform usual activities for at least one day, or accessed formal injury care, or both. These participants were identified through the Karonga HDSS key informant network embedded within the local community [17]. The second consisted of adult members of the general public without experience of significant injury. They were identified from households close to those of the first focus group participants. The third consisted of community leaders identified through the traditional authority network within the HDSS. Candidates were selected from those potentially eligible to cover a range of mechanism of injury (for the first FGD), gender, age, and location within the HDSS. Limiting to 3 FGDs was an a priori decision based on available resources and permissions obtained.

#### Participant recruitment

Two native Chitumbuka speaking research assistants (RAs) visited these individuals in their communities to explain the project using the participant information sheet at least 24 hours in advance of the discussion group meetings. These same RAs assisted in conducting the group discussion.

#### Discussion group conduct

Following initial training and practice, FGDs were facilitated by an RA, in Chitumbuka, following a translated discussion guide. Discussions took place at a building located centrally within the HDSS (Uliwa trading centre) convenient for participants. Present at each discussion workshop were two native Chitumbuka speaking employed RAs, one male, one female, trained and experienced in qualitative research within the specific community and two project researchers non-native speakers (JW a British male and ET a Nigerian female). Participants were not specifically known to the research team in advance of the study.

To help orientate participants to the subject of injuries, the RAs provided examples of physical injuries using the sentinel conditions (Figure ‎5‑1). Then they asked participants to describe, in order, potential barriers then facilitators to care-seeking (delay 1), reaching care (delay 2) or receiving quality care (delay 3) following injury within their community. To aid discussion “spidergrams” were created upon which the barriers and facilitators were placed. ‘Spidergrams’ are visual tools for identifying and analysing relationships. The spider’s body may be used to define the focus (in this case a conceptual delay) with the legs used to reflect factors relevant (in this case barriers or facilitators) [18]. Discussions lasted approximately 90 minutes. FGDs were audio-recorded and subsequently transcribed and translated into English by MEIRU trained staff. The “spidergrams” were photographed for use in analysis as field notes. Discussions continued until no additional factors acting as either a barrier or a facilitator to injury care were proposed; this was deemed to represent conceptual saturation for each discussion group.

Participants from the first FGD (injured in the past 12 months) were then invited to continue as participants in the photovoice study. There were otherwise no repeated interviews or discussions involving the other FGD participants. All participants from the first FGD all agreed to continue as participants in the photovoice study. The photovoice study is conceived as an extension of the community FGD study.

Photovoice participants were provided with a basic digital camera for use to return to the research team on completion. They participated in a training session on using the provided camera, basic principles of photography, the ethical implications of taking photos of people, the principles of photovoice, and the study aims [19]. Participants were asked to use the medium of photography to illustrate what they believe to be important barriers to seeking, reaching and receiving good quality healthcare after injury. Participants were provided with a printed training manual for reference and given one week to take the photos. Midway through the week the research team visited participants at home to ensure progress and identify and resolve any problems or misunderstandings. After one week, participants were visited and selected three images that best illustrated the barriers they wished to discuss and provided titles for the images. The research team printed these photos on A4 laminate paper. All the photovoice participants then reconvened together at the original meeting place for a follow-up discussion group the following day. During this follow up discussion participants were asked to explain the meaning behind each photo and why they took it and wish to highlight it. Participants discussed how each photo might be mapped to the Three Delays conceptual framework. Each photo was placed onto a Venn Diagram like structure with a location for each delay or overlapping delays (Figure ‎5‑4). Photovoice participants were asked to describe their experience of being involved in the study. This included any challenges encountered and any photos they would have like to take but couldn’t. The meeting was audio recorded. Where photos selected involved identifiable people, those individuals were identified, visited and specific written consent for use of the images sought. If individuals could not be located, then faces within the images were to be blurred to make the individuals unrecognisable. Translation and transcription of all audio recordings were conducted by trained native Chitumbuka speakers fluent in English. It was not possible to confirm transcriptions with participants for practical reasons.

### Analysis

Analysis of both the FGD and photovoice study data was conducted concurrently. Data analysis was conducted using the framework analytical method, including familiarisation; identifying a thematic framework; indexing; charting; and interpretation [20, 21]. It is well matched to this study's specific research question. It is suitable when time frames are limited, participant samples are clearly defined, and the issues of focus have been considered a priori. Such an analytical approach aligns well with the principles of Rapid Assessment [22, 23]. Although framework analysis may generate novel theories, the prime objective is to describe and deduce what is happening in a particular setting [24]. For the purpose of this study analysis the a priori framework was the Three Delays framed barriers to injury care generated by the Delphi expert consensus study [16]. This was used as a starting point for deductive analysis with scope for additional barriers and themes emerging through inductive analysis [24]. We conducted data coding using NVivo 12 software [25]. Discussion transcripts, photovoice images and “spidergrams” were all used to aid interpretation. Barrier themes additional to those from the Delphi study were generated. For this analysis, barriers and facilitators were consolidated to allow interpretation of reciprocal descriptions, i.e. a lack of something can be a barrier, but a presence of the same can be framed as a facilitator [26-28]. Discussions were constructed to explore both dimensions to encourage capturing as many important potential health system barriers as possible.

## Healthcare Worker Survey

### Aim

To establish local healthcare worker perceptions of each of the Three Delays', their relative importance and the barriers contributing to them.

### Data Collection

#### Survey Development

The healthcare worker survey was constructed de novo within a Three Delays framework. The survey was developed based upon the Delphi study findings using the identified conceptual barriers to injury care important to assess [16] and some barriers generated from the community FGDs. The survey was translated from English to Chitumbuka and back-translated to confirm accuracy and retention of meaning. The survey was piloted amongst four native Chitumbuka speaking healthcare workers not working at the included facilities and minor adjustments made to improve comprehension.

#### Identification of participants

Identified facilities were visited with the permission of the senior clinical authority at each location. All available staff members during the time of facility visits (e.g. not on leave) who had been involved in an injured person’s care in the preceding twelve months were eligible and approached to take part in the survey. Eligible participants included those staff groups who provide direct care and those indirectly involved, such as technical and administrative staff, who might share insight into barriers and delays experienced by injured persons [29].

The administered survey was conducted between July and October 2019 in Chitumbuka by two native speaking research assistants (both qualified Medical Assistants) at a convenient time and quiet location within the facility. Survey responses were collected onto electronic tablets using REDCap Mobile App and uploaded onto the REDCap [9] server database at the end of each facility visit. Each survey took between 30-60 minutes to complete.

The survey contained questions on sex, frequency of caring for an injured person (mutually exclusive categories of daily, weekly, monthly, quarterly and annually), the name of their place of work, job role (categorised as doctor, nurse, clinical officer, prehospital worker, medical assistant or other which was then specified), level of training in injury care (mutually exclusive categories of no formal training in the care of the injured, training received during primary healthcare qualification, post-qualification training through a course up to less than ten days in total, or significant post-qualification training including formal postgraduate qualifications, placements, fellowships or courses more than ten days) and time since last injury training (mutually exclusive categories of less than one year ago, between 1 and 3 years ago and more than 3 years ago).

Participants estimated which of Delays 1-3 affected the largest and smallest number of people, caused the most and least delays to those affected, were the easiest and most difficult to change to improve injury care, and overall which were the most and least important.

Participants were then asked to estimate how many patients (mutually exclusive categories of almost all patients (81-100%), more than half but not all patients (61-80%), about half (41-60%), some, but less than half (21-40%), few (1-20%) and none) experienced any delay seeking care, delay reaching care (specified as reaching care within 24, 12, 6, 2 and 1 hours), or any delay receiving care. Participants were asked to estimate typical time delays in seeking and receiving injury care (mutually exclusive categories of <1 hour, >1 but <2 hours, >2 but <4 hours, >4 but <6 hours, >6 but <12 hours, >12 but <24 hours and >24 hours). Participants were also asked to estimate how much harm came to patients who had experienced delays seeking care, delays reaching care (of between 1-4 hours and more than 4 hours), and delays receiving care, (mutually exclusive categories no or minimal harm, minor harm, slightly prolonged pain or suffering but very low risk of long term consequences, significant harm that poses a small or moderate risk to causing long term harm or a small risk to life and very significant harm, likely to cause high risk of long term harm or high risk to life).

Participants were presented with the Delphi [16] derived theoretical barriers to care for each delay (S1 Text Table 2). They were asked to put them in order (from most to least) of a) affecting the largest number and b) causing the longest amount of delay to those affected. Participants could also propose additional barriers using free text and indicate where they would place them in the above order. Participants were further asked to indicate which of all the barriers they considered the three most important overall from all conceptual delays.

### Outcome variables

Facility types were categorised as primary care or referral (secondary or tertiary care). Frequency of providing injury care was dichotomised to at least weekly and less than weekly, and injury care training level dichotomised into any postgraduate injury care training, including short courses and no postgraduate training. Job role was dichotomised to clinical care provider (including Doctors, Nurses, Clinical Officers, Medical Assistants and Ambulance care workers) and “other”. The conceptual delay affecting the second largest number of people, amount of delay, ease of change, and overall importance was deduced from the largest and smallest reported (as not asked directly).

Barriers ordered by participants were assigned a score corresponding to the order they were placed. Barriers affecting the first largest number of people were scored 1, those affecting the second largest number of people were scored 2 etc. A mean score across all participants was then calculated for each barrier for a) affecting the largest number of patients, b) causing the longest amount of delay, and c) a combined mean barrier score (a+b). Lower scores indicate more important barriers.

The primary outcome was participant perceptions of the importance of Delays 1-3 (estimates of the number affected at each delay stage, length of delays and harm associated with Delays 1-3). Secondary outcomes were the mean barrier scores and the number of participants reporting each barrier within the top 3 most important overall.

### Analysis

Participant characteristics, perceptions of aspects of importance of Delays 1-3, estimates of the number affected at each delay stage, length of delays and harm associated with Delays 1-3 were all described with counts and percentages. Barrier scores are described as mean scores. Counts describe each barrier reported within the top 3 most important overall. We reviewed free text responses proposing barriers and judged whether the proposed barriers were novel or could be subsumed into other categories.

S1 Text Table 2 List of barriers healthcare worker participants could select from, by Delay.

Legend - * denotes barrier was included after identification from concurrent community-based study.

| **Delay 1 (Seeking Care)** |
| --- |
| The perceived financial costs associated with seeking care are too great |
| Normal cultural behaviours delay seeking care such as gender roles, family responsibilities and requiring someone else's permission to seek care |
| People perceive that care is too difficult to physically access |
| People don't understand about health or available healthcare. |
| People perceive that available facility care is poor quality |
| There are delays in discovering injured people, including because of intoxication |
| People prefer traditional healers |
| People fear the consequences of helping an injured person, e.g. being accused of causing the injury* |
| **Delay 2 (Reaching Care)** |
| There is a lack of timely affordable emergency transport (formal or informal) |
| There is a large physical distance from place of injury to an appropriate healthcare facility |
| There is a lack of timely available prehospital emergency care (formal or informal/bystander) |
| There is a lack of accessible emergency assistance communication mechanism (e.g. emergency call centre) |
| There is a lack of emergency care service coordination, including bypassing unsuitable facilities or transferring between facilities |
| There is a lack of reliable uncongested roads with priority for emergency vehicles |
| **Delay 3 (Receiving Care)** |
| There is a lack of reliably available necessary physical resources (e.g. infrastructure, equipment and consumable material) |
| In regard to staffing, there is a lack of reliably available, suitably trained and motivated clinical staff |
| Specialist services needed for some injuries are not available in this area. |
| There is a lack of good quality, structured care processes for injured patients. |
| Lack of available means to safely and quickly transfer injured patients on to a more specialist facility* |
| In regard to patient demand, there is insufficient facility capacity to meet patient demand (e.g. overcrowding) |
| There is a lack of patient and family cooperation with care processes |
| Difficulties with timely payment for care |
| Need for unauthorised payments or gifts to healthcare staff to receive best available treatment. (e.g. corruption) * |

## Facility Assessment

### Aim

To describe the availability of resources within the health system’s facilities relative to those deemed essential by the World Health Organisation’s Essential Trauma Care checklist (WHO ETC).

### Data Collection:

As with all facility-based study components, permission was sought from the lead facility clinician to undertake the assessment. The lead clinician identified the most suitable person or persons to provide the answer for each assessment tool question. We inspected the facility accompanied by the facility staff member(s), to verify the presence of items on the day of the visit. Depending on the facility size, this took between 1-5 hours per facility. All 11 facilities identified as likely to care for an injured person within the Karonga HDSS were included. Facility assessments occurred from July to October 2019, concurrently with the other facility-based study components (process mapping, clinical vignettes and healthcare worker (HCW) surveys).

The data collection instrument containing the questions used is found in Unpublished Methods Supplementary Material Appendix 1. Due to the large size of this instrument the questions will not be further detailed here.

### Outcome variables:

For each facility the proportion of physical resource (equipment and medication), available 90% of the time or more, classified by the WHO ETC as essential for a basic facility, general practitioner staffed hospital and specialist staffed hospital respectively were calculated. This was the primary study outcome. We determined a list of resources from the modified WHO ETC checklist necessary to manage each of the four sentinel injury conditions (blunt chest injury, penetrating abdominal wound, severe head injury and open lower limb fracture). This list was independently checked by another trauma care practitioner experienced in LMIC trauma health systems research. Any disagreements were discussed until consensus was achieved. Each item was weighted equally, and the proportion of total items available 90% of the time for each scenario was calculated per facility. This was the secondary study outcome. Facilities types were categorised as primary, secondary or tertiary.

### Data Analysis:

The generic and trauma specific facility assessment findings are described with counts and percentages for categorical data, and medians and ranges for continuous non-parametric data. The primary and secondary outcomes are described as mean percentage resource availability according to facility type.

**References for currently unpublished methods supplementary material.**

1. Sethi D HS, McGee K, Peden M, et al. Guidelines for Conducting Community Surveys on Injuries and Violence. World Health Organisation, , 2004.

2. Tayeb SE, Abdalla S, Mørkve O, Heuch I, Van den Bergh G. Injuries in Khartoum state, the Sudan: a household survey of incidence and risk factors. Int. J. Injury Control Safe. Promot. 2014;21(2):144-53. doi: 10.1080/17457300.2013.792283.

3. Mock CN, Abantanga F, Cummings P, Koepsell TD. Incidence and outcome of injury in Ghana: a community-based survey. Bull. World Health Organ. 1999;77(12):955-64. Epub 2000/02/19. PubMed PMID: 10680242; PubMed Central PMCID: PMCPMC2557773.

4. Moshiro C, Heuch I, Åstrøm AN, Setel P, Hemed Y, Kvåle G. Injury morbidity in an urban and a rural area in Tanzania: an epidemiological survey. BMC Public Health. 2005;5(1):11. doi: 10.1186/1471-2458-5-11.

5. El Tayeb S, Abdalla S, Van den Bergh G, Heuch I. Use of healthcare services by injured people in Khartoum State, Sudan. Int Health. 2015;7(3):183-9. Epub 2014/09/11. doi: 10.1093/inthealth/ihu063. PubMed PMID: 25205849; PubMed Central PMCID: PMCPMC4427533.

6. Edem IJ, Dare AJ, Byass P, D’Ambruoso L, Kahn K, Leather AJM, et al. External injuries, trauma and avoidable deaths in Agincourt, South Africa: a retrospective observational and qualitative study. BMJ Open. 2019;9(6):e027576. doi: 10.1136/bmjopen-2018-027576.

7. Mock C, Ofosu A, Gish O. Utilization of district health services by injured persons in a rural area of Ghana. The International Journal of Health Planning and Management. 2001;16(1):19-32. doi: 10.1002/hpm.607.

8. Spangenberg K, Mock C. Utilization of health services by the injured residents in Kumasi, Ghana. Int J Inj Contr Saf Promot. 2006;13(3):194-6. Epub 2006/09/01. doi: 10.1080/17457300500294455. PubMed PMID: 16943164.

9. Harris PA, Taylor R, Thielke R, Payne J, Gonzalez N, Conde JG. Research electronic data capture (REDCap)—A metadata-driven methodology and workflow process for providing translational research informatics support. Journal of Biomedical Informatics. 2009;42(2):377-81. doi: <https://doi.org/10.1016/j.jbi.2008.08.010>.

10. Crampin AC, Dube A, Mboma S, Price A, Chihana M, Jahn A, et al. Profile: the Karonga Health and Demographic Surveillance System. Int. J. Epidemiol. 2012;41(3):676-85. Epub 2012/06/21. doi: 10.1093/ije/dys088. PubMed PMID: 22729235.

11. Chihana M, Floyd S, Molesworth A, Crampin AC, Kayuni N, Price A, et al. Adult mortality and probable cause of death in rural northern Malawi in the era of HIV treatment. Trop. Med. Int. Health. 2012;17(8):e74-e83. doi: 10.1111/j.1365-3156.2012.02929.x.

12. Network. I. INDEPTH Network revised verbal autopsy questionnaire (Revised 2003): INDEPTH Network; 2003 [accessed 17 Aug 2020]. Available from: <http://www.indepth-network.org/resources/indepth-standardized-verbal-autopsy-questionnaire>.

13. Chasimpha S, McLean E, Chihana M, Kachiwanda L, Koole O, Tafatatha T, et al. Patterns and risk factors for deaths from external causes in rural Malawi over 10 years: a prospective population-based study. BMC Public Health. 2015;15(1):1036. doi: 10.1186/s12889-015-2323-z.

14. Glynn JR, Calvert C, Price A, Chihana M, Kachiwanda L, Mboma S, et al. Measuring causes of adult mortality in rural northern Malawi over a decade of change. Glob Health Action. 2014;7(1):23621. doi: 10.3402/gha.v7.23621.

15. D’Ambruoso L, Kahn K, Wagner RG, Twine R, Spies B, van der Merwe M, et al. Moving from medical to health systems classifications of deaths: extending verbal autopsy to collect information on the circumstances of mortality. Glob Health Res Policy. 2016;1(1):2. doi: 10.1186/s41256-016-0002-y.

16. Whitaker J, Nepogodiev D, Leather A, Davies J. Assessing barriers to quality trauma care in low and middle-income countries: A Delphi study. Injury. 2019. Epub 2019/12/31. doi: 10.1016/j.injury.2019.12.035. PubMed PMID: 31883865.

17. Dube A, Price A, Jahn A, Baschieri A, Molesworth A, Zaba B, et al. Profile: The Karonga Health and Demographic Surveillance System. Int. J. Epidemiol. 2012;41(3):676-85. doi: 10.1093/ije/dys088.

18. Rene Loewenson ACL, Christer Hogstedt, Lucia D’Ambruoso, Zubin Shroff. . Participatory Action Research in health systems, A Methods Reader. . Alliance for Health Policy and Systems Research, 2014.

19. Sutton-Brown CA. Photovoice: A Methodological Guide. Photography and Culture. 2014;7(2):169-85. doi: 10.2752/175145214X13999922103165.

20. Srivastava AT, Stanley Bruce. **Framework analysis: A qualitative methodology for applied policy research**. Journal of Administration and Governance. 2009;4:72-9.

21. Gale NK, Heath G, Cameron E, Rashid S, Redwood S. Using the framework method for the analysis of qualitative data in multi-disciplinary health research. BMC medical research methodology. 2013;13:117-. doi: 10.1186/1471-2288-13-117. PubMed PMID: 24047204.

22. Beran D, Yudkin JS, de Courten M. Assessing health systems for type 1 diabetes in sub-Saharan Africa: developing a 'Rapid Assessment Protocol for Insulin Access'. BMC Health Serv. Res. 2006;6:17. Epub 2006/03/01. doi: 10.1186/1472-6963-6-17. PubMed PMID: 16504123; PubMed Central PMCID: PMCPMC1402284.

23. Whitaker J, Donohoe N, Denning M, Poenaru D, Guadagno E, Leather AJM, Davies JI. Assessing trauma care systems in low-income and middle-income countries: a systematic review and evidence synthesis mapping the Three Delays framework to injury health system assessments. BMJ Glob Health. 2021;6(5):e004324. doi: 10.1136/bmjgh-2020-004324.

24. Ritchie, J. & Spencer, L. 1994. Qualitative data analysis for applied policy research by Jane Ritchie and Liz Spencer in A. Bryman and R. G. Burgess [eds.] ‘Analysing qualitative data’, (pp.173-194). London: Routledge.

25. NVivo Qualitative Data Analysis Software. QSR International; 1999.

26. Oliver K, Innvar S, Lorenc T, Woodman J, Thomas J. A systematic review of barriers to and facilitators of the use of evidence by policymakers. BMC Health Serv. Res. 2014;14:2-. doi: 10.1186/1472-6963-14-2. PubMed PMID: 24383766.

27. Feyissa GT, Woldie M, Munn Z, Lockwood C. Exploration of facilitators and barriers to the implementation of a guideline to reduce HIV-related stigma and discrimination in the Ethiopian healthcare settings: A descriptive qualitative study. PLoS One. 2019;14(5):e0216887. doi: 10.1371/journal.pone.0216887.

28. Driessen MT, Groenewoud K, Proper KI, Anema JR, Bongers PM, van der Beek AJ. What are possible barriers and facilitators to implementation of a Participatory Ergonomics programme? Implement Sci. 2010;5(1):64. doi: 10.1186/1748-5908-5-64.

29. Joseph B, Joseph M. The health of the healthcare workers. Indian J. Occup. Environ. Med. 2016;20(2):71-2. doi: 10.4103/0019-5278.197518. PubMed PMID: 28194078.

Unpublished Methods Supplementary Material Appendix 1 - Facility assessment survey tool with survey data entry codes used for data collection.

MALAWI EPIDEMIOLOGY AND INTERVENTION RESEARCH UNIT

MULTI-METHOD EVALUATION OF THE TRAUMA CARE HEALTH SYSTEM SERVING KARONGA, MALAWI

Facility Assessment

This facility assessment has been developed from World Health Organisation’s (WHO) Service Availability and Readiness Assessment (SARA), the WHO Essential Trauma Care (ETC) guidelines checklist and the African Federation of Emergency Medicine Emergency Care Assessment Tool (ECAT). It is intended to be administered by a member of the research team, with items confirmed through inspection of the health facility, where possible. It should take no more than 1 working day to complete, depending on staff availability.

| Section 1. Facility Overview | | | |
| --- | --- | --- | --- |
| 1.1 | Name of facility | Free Text…….. | FACNAM |
| 1.2 | Location of facility | Free Text…….. | FACLOC |
| 1.3 | Region | Free Text…….. | FACREG |
| 1.4 | District | Free Text…….. | FACDIS |
| 1.5 | Type of facility | Specialist Trauma Hospital = 1  Referral hospital = 2  District hospital = 3  Health clinic or smaller = 4  Rural Hospital = 5  Other (specify)………………….. = 96 | FACTYP |
| 1.6 | Managing Authority | Government / public = 1  NGO / not for profit = 2  Private / for profit = 3  Mission / faith based = 4  Other (specify)………………..=96 | FACAUT |
| 1.7 | Urban/Rural | Urban = 1  Rural = 2  Missing =99 | FACUR |
| 1.8 | Outpatient only | Yes = 1  No = 0  Missing = 99 | FACOP |
| 1.9 | Excluding any delivery beds, how many overnight/inpatient beds in total does this facility have, both for adults and children? | Numeric = | FACBED |
| 1.10 | Of the overnight/inpatient beds in this facility, how many are dedicated maternity beds? | Numeric = | FACMAT |

| Section 2. Staffing | | | |
| --- | --- | --- | --- |
| I have a few questions on staffing for this facility. Please tell me how many staff with each of the following qualifications are currently assigned to, employed by, or seconded to this facility. Please count each staff member only once, on the basis of the highest technical or professional qualification. For doctors, I would also like to know, of the total number, how many are part-time in this facility: | | | |
|  | Role |  |  |
| 2.1 | Generalist (non-specialist) medical doctors   1. Total number including part time 2. Number part time only 3. Number involved in the care of the injured | Numeric =  Numeric =  Numeric = | SGENTOT  SGENPT  SGENINJ |
| 2.2 | Specialist medical doctors   1. Total number including part time 2. Number part time only 3. Number involved in the care of the injured | Numeric =  Numeric =  Numeric = | SSPECTOT  SSPECPT  SSPECINJ |
| 2.3 | Non-physician clinicians/paramedical professionals   1. Total number including part time 2. Number part time only 3. Number involved in the care of the injured | Numeric =  Numeric =  Numeric = | SNPCTOT  SNPCPT  SNPCINJ |
| 2.4 | Nursing professionals   1. Total number including part time 2. Number part time only 3. Number involved in the care of the injured | Numeric =  Numeric =  Numeric = | SNURTOT  SNURPT  SNUINJ |
| 2.5 | Midwifery professionals   1. Total number including part time 2. Number part time only 3. Number involved in the care of the injured | Numeric =  Numeric =  Numeric = | SMIDTOT  SMIDPT  SMIDINJ |
| 2.6 | Laboratory technicians (medical and pathology)   1. Total number including part time 2. Number part time only 3. Number involved in the care of the injured | Numeric =  Numeric =  Numeric = | SLABTOT  SLABPT  SLABINJ |
| 2.7 | Community health workers   1. Total number including part time 2. Number part time only 3. Number involved in the care of the injured | Numeric =  Numeric =  Numeric = | SCOMTOT  SCOMPT  SCOMINJ |
| 2.8 | Pharmacists   1. Total number including part time 2. Number part time only 3. Number involved in the care of the injured | Numeric =  Numeric =  Numeric = | SPHATOT  SPHAPT  SPHAINJ |

| Section 3. General Services | | | |
| --- | --- | --- | --- |
| 3.1 | On average, how many hours per day is this facility open? | < 4 hours = 1  5-8 hours = 2  9-16 hours = 3  17-23 hours = 4  24 hours = 5  Missing = 99 | FACOPHR |
| 3.2 | What is the ***most commonly used*** source of water for the facility ***at this time***? | No Water Source = 0  Piped into facility = 1  Piped onto facility grounds = 2  Public tap/standpipe = 3  Tube well/borehole = 4  Protected dug well = 5  Unprotected dug well = 6  Protected spring = 7  Unprotected spring = 8  Rainwater collection = 9  Bottled water = 10  Cart w/small tank/drum = 11  Tanker truck = 12  Surface water = 13  Other = 96  …………………………….  Don’t know = 98  Missing = 99 | FACWATSOU |
| 3.3 | Is water available from this source on facility premises? | Yes, inside the facility = 1  Yes, within the ground of the facility = 2  No, outside the facility grounds = 0 | FACWATAV |
|  | | | |

| 3.4 | Is there a toilet (latrine) on premises in functioning condition that is accessible for general outpatient client use? IF YES: What type of toilet?  If multiple toilets are available, consider the most modern type.  Observe that the toilet (latrine) is accessible (unlocked or key available) and functioning | Flush toilet = 1  Ventilated improved pit latrine (VIP) = 2  Pit latrine with slab = 3  Pit latrine without slab/open pit = 4  Composting toilet = 5  Bucket = 6  Hanging toilet / hanging latrine = 7  No facilities on premises/bush/field = 0  Don’t know = 98  Missing = 99 | FACTOIL |
| --- | --- | --- | --- |
| 3.5 | During the past 7 days, was electricity available at all times from the main or any backup source when the facility was open for services? | Always available (no interruptions) = 1  Often available (interruptions of less than 2 hours per day) = 2  Sometimes available (frequent or prolonged interruptions of more than 2 hours per day) = 3  Don’t know = 98  Missing = 99 | FACELEC |
| 3.6 | Does this facility have a functional ambulance or other vehicle for emergency transportation for clients that is stationed at this facility or operates from this facility? | Yes = 1  No = 0  Don’t know = 98  Missing = 99 | AMBSTA |
| 3.7 | Does this facility have access to an ambulance or other vehicle for emergency transport for clients that is stationed at another facility or that operates from another facility in near proximity? | Yes = 1  No = 0  Don’t know = 98  Missing = 99 | AMBACC |
| 3.8 | Is fuel for the ambulance or other emergency vehicle available today? | Yes = 1  No = 0  Don’t know = 98  Missing = 99 | AMBFUE |
| 3.9 | Is the ambulance equipped to treat injured persons | Yes = 1  No = 0  Don’t know = 98  Missing = 99 | AMBEQUINJ |
| 3.10 | Are staff on the ambulance able to provide emergency care to injured patients? | Yes = 1  No = 0  Don’t know = 98  Missing = 99 | AMBSTAINJ |
| 3.11 | How often does the ambulance transfer injured patients from the community to this hospital? | Most days = 1  At least once per week but not most days = 2  At least once a month but not every week = 3  At least every 3 months, but not every month = 4  At least every year but not every 3 months = 5  Less than every year = 6  Don’t know = 98  Missing = 99 | AMBTFCOMM |
| 3.12 | How often does the ambulance transfer injured patients from this hospital to another more specialist facility? | Most days = 1  At least once per week but not most days = 2  At least once a month but not every week = 3  At least every 3 months, but not every month = 4  At least every year but not every 3 months = 5  Less than every year = 6  Don’t know = 98  Missing = 99 | AMBTFHOSP |
| 3.13 | Does this facility have a functioning land line telephone that is available to call outside at all times client services are offered?  Clarify that if facility offers 24-hour emergency services, then this refers to 24-hour availability. | Yes = 1  No = 0  Don’t know = 98  Missing = 99 | FACLLTEL |
| 3.14 | Does this facility have a functioning cellular telephone or a private cellular phone that is supported by the facility? | Yes = 1  No = 0  Don’t know = 98  Missing = 99 | FACMOBTEL |
| 3.15 | Does this facility have a functioning short-wave radio for radio calls? | Yes = 1  No = 0  Don’t know = 98  Missing = 99 | FACSWRAD |
| 3.16 | Does this facility have a functioning computer? | Yes = 1  No = 0  Don’t know = 98  Missing = 99 | FACCOMP |
| 3.17 | Is there access to email or internet within the facility today? | Yes = 1  No = 0  Don’t know = 98  Missing = 99 | FACINTERNET |
| 3.18 | Does this facility offer any surgical services (including minor surgery such as suturing, wound debridement etc) or caesarean section? | Yes = 1  No = 0  Don’t know = 98  Missing = 99 | FACSURG |

| Section 4. Trauma Care Overview | | | | | | | | | | | | |
| --- | --- | --- | --- | --- | --- | --- | --- | --- | --- | --- | --- | --- |
| 4.1 | | Not including patient medical records, does your facility keep a specific registry of patients treated with injury? If so is it electronic or hard copy? | | | Yes electronic = 1  Yes paper based = 2  No = 3  Don’t know = 98  Missing = 99 | | | | | FACTRREG | | |
| 4.2 | | How often does your facility treat an injured person? | | | Most days = 1  At least once per week but not most days = 2  At least once a month but not every week = 3  At least every 3 months, but not every month = 4  At least every year but not every 3 months = 5  Less than every year = 6  Don’t know = 98  Missing = 99 | | | | | FACINJFREQ | | |
| 4.3 | | How often does a person die, because of injury, in this facility? | | | Most days = 1  At least once per week but not most days = 2  At least once a month but not every week = 3  At least every 3 months, but not every month = 4  At least every year but not every 3 months = 5  Less than every year = 6  Don’t know = 98  Missing = 99 | | | | | FACINJMORT | | |
| 4.4 | | How often is an injured person brought to hospital already dead because of an injury? | | | Most days = 1  At least once per week but not most days = 2  At least once a month but not every week = 3  At least every 3 months, but not every month = 4  At least every year but not every 3 months = 5  Less than every year = 6  Don’t know = 98  Missing = 99 | | | | | PHINJMORT | | |
| 4.5a | | Does the facility have any policies for the management of injured persons? | | | Yes = 1  No = 0  Don’t know = 98  Missing = 99 | | | | | FACINJPOL | | |
| 4.5b | |  | Please describe or demonstrate the policies or guidelines in place if applicable | | Free text……………….. | | | | | FACPOLTXT | | |
| 4.6a | | Does the facility have a designated team to manage injured persons? | | | Yes = 1  No = 0  Don’t know = 98  Missing = 99 | | | | | FACINJTM | | |
| 4.6b | |  | If Yes, what is the team comprised of? | | Free Text Response | | | | | INJTMPAX | | |
| 4.6c | |  | When was the team last mobilized / activated? | | This week = 1  This month but not this week = 2  In the last 3 months but not the last month = 3  In the last year but not in the last 3 months = 4  Not within the last year = 5  Don’t know = 98  Missing = 99 | | | | | INJTMACT | | |
| 4.7 | | Are facility staff required to undergo specific training for the care of the injured? | | | Yes = 1  No = 0  Don’t know = 98  Missing = 99 | | | | | INJSTFTRG | | |
|  | | Regarding staff training, please indicate the **number of staff** in each role, who have the following training in the care of the injured | | | | | | | | | | |
| 4.8a | | No formal training in the care of injured patients   1. Generalist (non-specialist) medical doctors 2. Specialist medical doctors 3. Non-physician clinicians/paramedical professionals 4. Nursing professionals 5. Community health workers | | | Numeric =  Numeric =  Numeric =  Numeric =  Numeric = | | | | | GMNLTRG  SPCNLTRG  NPCNLTRG  NSNLTRG  CHWNLTRG | | |
| 4.8b | | Training in the care of the injured during primary healthcare qualification   1. Generalist (non-specialist) medical doctors 2. Specialist medical doctors 3. Non-physician clinicians/paramedical professionals 4. Nursing professionals 5. Community health workers | | | Numeric =  Numeric =  Numeric =  Numeric =  Numeric = | | | | | GMPQTRG  SPCPQTRG  NPCPQTRG  NSPQTRG  CHWPQTRG | | |
| 4.8c | | Post qualification training in the care of the injured through a course equivalent to less than 10 days in total   1. Generalist (non-specialist) medical doctors 2. Specialist medical doctors 3. Non-physician clinicians/paramedical professionals 4. Nursing professionals 5. Community health workers | | | Numeric =  Numeric =  Numeric =  Numeric =  Numeric = | | | | | GMPGTRG  SPCPGTRG  NPCPGTRG  NUPGTRG  CHWPGTRG | | |
| 4.8d | | Significant post qualification training in the care of the injured including formal post graduate educational qualifications, specialist placements or fellowships, or courses greater than 10 days in length   1. Generalist (non-specialist) medical doctors 2. Specialist medical doctors 3. Non-physician clinicians/paramedical professionals 4. Nursing professionals 5. Community health workers | | | Numeric =  Numeric =  Numeric =  Numeric =  Numeric = | | | | | GMEXPTRG  SPCEXPTRG  NPCEXPTRG  NUEXPTRG  CHWEXPTRG | | |
| 4.9 | | Has your facility conducted a review of injury related deaths? | | | Never = 1  In the last 5 years = 2  In the last year = 3  In the last 6 months = 4  In the last 3 months = 5  In the last month = 6  Don’t know = 98  Missing = 99 | | | | | FACINJREV | | |
| 4.10a | | Does your facility have any routine quality improvement activity aiming to improve the care of injured persons? | | | Yes = 1  No = 0  Don’t know = 98  Missing = 99 | | | | | FACINJQI | | |
| 4.10b | |  | If Yes, what is it  ………………………… | | Free Text | | | | | INJQITXT | | |
| 4.10c | |  | When was it last conducted? | | Never = 1  In the last 5 years = 2  In the last year = 3  In the last 6 months = 4  In the last 3 months = 5  In the last month = 6  Don’t know = 98  Missing = 99 | | | | | INJQILAST | | |
| Section 5. Blood Transfusion Capability | | | | | | | | | | | |  |
| 5.1 | Does this facility offer blood transfusion services? | | | Yes = 1  No = 0 SKIP TO SECTION 6  Don’t know = 98  Missing = 99 | | | | | | | | FACBLDTF |
| 5.2 | Have there been any interruptions in blood availability during the past 3 months? | | | Yes = 1  No = 0  Don’t know = 99  Missing = 99 | | | | | | | | BLDINT |
| 5.3 | Does this facility obtain blood from a national or regional blood centre? | | | Yes = 1  No = 0  Don’t know = 98  Missing = 99 | | | | | | | | BLDCTR |
| 5.4 | Does this facility obtain ANY blood from sources other than the national or regional blood centre? | | | Yes = 1  No = 0  Don’t know = 98  Missing = 99 | | | | | | | | BLDSRC |
| 5.5 | Does any place in this facility do blood screening for infectious diseases prior to transfusion? | | | Yes = 1  No = 0  Don’t know = 98  Missing = 99 | | | | | | | | BLDSCRN |
|  | Please tell me if the blood that is transfused in the facility is "always", "sometimes", ”rarely”, or "never" screened for any of the following infectious diseases: | | | ALWAYS | | SOMETIMES | RARELY | NEVER | DON’T KNOW | | MISSING |  |
| 5.6a | Human Immunodeficiency Virus | | | 1 | | 2 | 3 | 4 | 98 | | 99 | BLDHIV |
| 5.6b | Syphilis | | | 1 | | 2 | 3 | 4 | 98 | | 99 | BLDSYP |
| 5.6c | Hepatitis B | | | 1 | | 2 | 3 | 4 | 98 | | 99 | BLDHPB |
| 5.6d | Hepatitis C | | | 1 | | 2 | 3 | 4 | 98 | | 99 | BLDHPC |
| 5.7 | Does this facility have a refrigerator available and functioning in this service area for the storage of blood? | | | Available and functional = 1  Available not functional = 2 **SKIP to 5.11**  Available don't know if functioning = 3  Not available = 0 **SKIP to 5.11**  Don’t know = 98 **SKIP to 5.11**  Missing = 99 **SKIP to 5.11** | | | | | | | | BLDFRG |
| 5.8 | Is the temperature of the refrigerator monitored at least once every 24 hours? | | | Yes, log observed = 1  Yes, log reported not seen = 2  No = 0  Don’t know = 98  Missing = 99 | | | | | | | | FRGMNT |
| 5.9 | Has the temperature log been completed for the last 30 days? | | | Yes = 1  Yes, partially = 2  No = 3  Don’t know = 98  Missing = 99 | | | | | | | | BLDTMP |
| 5.10 | Has the temperature been out of the range 2 to 6 degrees Celsius inclusive in the last 30 days? | | | Observed in range = 1  Reported in range but not seen = 2  Out of range = 3  Record not available = 4  Don’t know = 98  Missing = 99 | | | | | | | | TMPRNG |
| 5.11 | Do you have any guidelines on the appropriate use of blood and safe transfusion practices? | | | Yes, observed = 1  Yes, reported not seen = 2  No = 3  Don’t know = 98  Missing = 99 | | | | | | | | BLDGL |
| 5.12 | Have any provider(s) of blood transfusion services received any training in the appropriate use of blood and safe transfusion practices in the last two years? | | | Yes = 1  No = 0  Don’t know = 98  Missing = 99 | | | | | | | | BLDTRG |

| Section 6. Trauma Care Equipment | | | | | | | | | |
| --- | --- | --- | --- | --- | --- | --- | --- | --- | --- |
|  | Regarding equipment important in the care of the injured: : | A - Is this item present today?  YES = 1  NO = 0  Don’t know = 98  Missing = 99 | | B - If yes - Is a suitably trained individual able to use this item to care for the injured >90% of the time?  YES = 1  NO = 0  Don’t know = 98  Missing = 99 | | C - If Yes to B - When was this item last used to treat a patient ?  Within the last week = 1  Within the last month = 2  Within the last 3 months = 3  Within the last 6 months – 4  Within the last year = 5  More than 1 year ago = 6  Never = 7  Not applicable = 97  Don’t know = 98  Missing = 99 | | D - If No to B - what is the reason? – tick all that apply  Lack of staff availability = 1  Lack of staff training = 2  Lack of staff motivation = 3  Lack of specialist services = 4  Lack of infrastructure = 5  Lack of equipment = 6  Lack of consumable material = 7  Lack of patient and family cooperation with care processes = 8  Lack of care processes such as policies and service organisation = 9  Difficulties with payment for care = 10  Insufficient facility capacity – overcrowding. = 11  Other (specify)………… = 12  Not applicable = 97  Don’t know = 98  Missing = 99 | |
| 6.1 | Oral or nasal airway | A | ONAA | B | ONAB | C | ONAC | D | ONAD |
| 6.2 | Functioning Suction device with tubing and tip | A | SUCA | B | SUCB | C | SUCC | D | SUCD |
| 6.3 | Laryngoscope | A | LARA | B | LARB | C | LARC | D | LARD |
| 6.4 | Endotracheal tube | A | ETTA | B | ETTB | C | ETTC | D | ETTD |
| 6.5 | Bag-valve-mask | A | BVMA | B | BVMB | C | BVMC | D | BVMD |
| 6.6 | Magill forceps | A | MAGA | B | MAGB | C | MAGC | D | MAGD |
| 6.7 | Capnography | A | CAPA | B | CAPB | C | CAPC | D | CAPD |
| 6.8 | Oxygen supply | A | O2SA | B | O2SB | C | O2SC | D | O2SD |
| 6.9 | Nasal prongs, face mask, associated tubing | A | NFMA | B | NFMB | C | NFMC | D | NFMD |
| 6.10 | Needle & syringe | A | NSYA | B | NSYB | C | NSYC | D | NSTD |
| 6.11 | Chest tubes with Underwater seal bottle (or equivalent) | A | ICDA | B | ICDB | C | ICDC | D | ICDD |
| 6.12 | Pulse oximetry | A | POXA | B | POXB | C | POXC | D | POXD |
| 6.13 | Mechanical ventilator | A | MEVA | B | MEVB | C | MEVC | D | MEVD |
| 6.14 | Clock or watch with second hand | A | CLKA | B | CLKB | C | CLKC | D | CLKD |
| 6.15 | Stethoscope | A | STEA | B | STEB | C | STEC | D | STED |
| 6.16 | Blood pressure (BP) cuff | A | BPCA | B | BPCB | C | BPCC | D | BPCD |
| 6.17 | Gauze and bandages | A | GZEA | B | GZEB | C | GZEC | D | GZED |
| 6.18 | Arterial tourniquet | A | ATQA | B | ATQB | C | ATQC | D | ATQD |
| 6.19 | Crystalloid | A | CRYA | B | CRYB | C | CRYC | D | CRYD |
| 6.20 | Colloids | A | COLA | B | COLB | C | COLC | D | COLD |
| 6.21 | Intravenous infusion set (lines and cannulas) | A | IVIA | B | IVIB | C | IVIC | D | IVID |
| 6.22 | Intraosseous access device | A | IODA | B | IODB | C | IODC | D | IODD |
| 6.23 | Central venous lines | A | CVCA | B | CVCB | C | CVCC | D | CVCD |
| 6.24 | Urinary catheter with collection bag | A | URCA | B | URCB | C | URCC | D | URCD |
| 6.25 | Electronic cardiac monitoring | A | ECMA | B | ECMB | C | ECMC | D | ECMD |
| 6.26 | Monitor for central venous pressure | A | CVPA | B | CVPB | C | CVCC | D | CVCD |
| 6.27 | Laboratory facilities for haemoglobin or  haematocrit | A | LHBA | B | LHBB | C | LHBC | D | LHBD |
| 6.28 | Laboratory facilities for electrolytes | A | LUEA | B | LUEB | C | LUEC | D | LUED |
| 6.29 | Laboratory facilities for lactate | A | LACA | B | LACB | C | LACC | D | LACD |
| 6.30 | Laboratory facilities for arterial blood gases | A | ABGA | B | ABGB | C | ABGC | D | ABGD |
| 6.31 | Vasopressors | A | VASA | B | VASB | C | VASC | D | VASD |
| 6.32 | Nasogastric (NG) tube | A | NGTA | B | NGTB | C | NGTC | D | NGTD |
| 6.33 | Thermometer | A | THEA | B | THEB | C | THEC | D | THED |
| 6.34 | Fluid warmers | A | FLWA | B | FLWB | C | FLWC | D | FLWD |
| 6.35 | Weighing scale for children | A | WSCA | B | WSCB | C | WSCC | D | WSCD |
| 6.36 | Intra Cranial Pressure measurement | A | ICPA | B | ICPB | C | ICPC | D | ICPD |
| 6.37 | Computerised Tomography scan | A | CTSA | B | CTSB | C | CTSC | D | CTSD |
| 6.38 | Burr holes drill or other suitable equipment | A | BURA | B | BURB | C | BURC | D | BURD |
| 6.39 | Endoscopy | A | ENDA | B | ENDB | C | ENDC | D | ENDD |
| 6.40 | Angiography | A | ANGA | B | ANGB | C | ANGC | D | ANGD |
| 6.41 | Intercostal or intrapleural block | A | ICBA | B | ICBB | C | ICBC | D | ICBD |
| 6.42 | Epidural analgesia | A | EPIA | B | EPIB | C | EPIC | D | EPID |
| 6.43 | Equipment for thoracotomy | A | THOA | B | THOB | C | THOC | D | THOD |
| 6.44 | Ultrasonography | A | USSA | B | USSB | C | USSC | D | USSD |
| 6.45 | Equipment for laparotomy | A | LAPA | B | LAPB | C | LAPC | D | LAPD |
| 6.46 | Basic immobilization equipment, slings and splints | A | SPLA | B | SPLB | C | SPLC | D | SPLD |
| 6.47 | Spine board | A | SPBA | B | SPBB | C | SPBC | D | SPBD |
| 6.48 | Pelvic binder | A | PBIA | B | PBIB | C | PBIC | D | PBID |
| 6.49 | Skin traction | A | TRAA | B | TRAB | C | TRAC | D | TRAD |
| 6.50 | Skeletal traction | A | SKTA | B | SKTB | C | SKTC | D | SKTD |
| 6.51 | Operative wound management | A | OWMA | B | OWMB | C | OWMC | D | OWMD |
| 6.52 | External fixation (or its functional equivalent: pins & plaster) | A | EXFA | B | EXFB | C | EXFC | D | EXFD |
| 6.53 | Internal fixation | A | IFXA | B | IFXB | C | IFXC | D | IFXD |
| 6.54 | X-ray | A | XRYA | B | XRYB | C | XRYC | D | XRYD |
| 6.55 | Portable X-ray | A | XRPA | B | XRPB | C | XRPC | D | XRPD |
| 6.56 | Image intensification | A | IMIA | B | IMIB | C | IMIC | D | IMID |
| 6.56 | Hard cervical collar | A | HCCA | B | HCCB | C | HCCC | D | HCCD |
| 6.57 | Magnetic Resonance Imaging | A | MRIA | B | MRIB | C | MRIC | D | MRID |
| 6.58 | Surgical treatment of spinal injury | A | SPIA | B | SPIB | C | SPIC | D | SPID |
| 6.59 | Sterile dressings | A | SDRA | B | SDRB | C | SDRC | D | SDRD |
| 6.60 | Topical antibiotic dressings | A | TADA | B | TADB | C | TADC | D | TADD |
| 6.61 | Skin graft equipment | A | SGEA | B | SGEB | C | SGEC | D | SGED |
| 6.62 | Reconstructive surgery | A | RCSA | B | RCSB | C | RCSC | D | RCSD |
| 6.63 | Tetanus prophylaxis (toxoid or antiserum) | A | TETA | B | TETB | C | TETC | D | TETD |
| 6.64 | Physiotherapy | A | PHYA | B | PHYB | C | PHYC | D | PHYF |
| 6.65 | Occupational therapy | A | OCTA | B | OCTB | C | OCTC | D | OCTD |
| 6.66 | Prosthetics | A | PROA | B | PROB | C | PROC | D | PROD |
| 6.67 | Psychological counseling | A | PSYA | B | PSYB | C | PSYC | D | PSYD |
| 6.68 | Neuropsychology for cognitive dysfunction | A | NPYA | B | NPYB | C | NPYC | D | NPYD |
| 6.69 | Speech pathology / therapy | A | SPTA | B | SPTB | C | SPTC | D | SPTD |
| 6.70 | Physical medicine and rehabilitation specialist-level care | A | RHBA | B | RHBB | C | RHBC | D | RHBD |
| 6.71 | Specialized rehabilitative nursing | A | RBNA | B | RBNB | C | RBNC | D | RBND |
| 6.72 | Torch (flashlight) | A | TCHA | B | TCHB | C | TCHC | D | TCHD |
| 6.73 | Contrast radiography (barium / gastrograffin) | A | CRAA | B | CRAB | C | CRAC | D | CRAD |
| 6.74 | Nuclear medicine | A | NMDA | B | NMDB | C | NMDC | D | NMDD |
| 6.75 | Measure Glucose | A | BMMA | B | BMMB | C | BMMC | D | BMMD |
| 6.76 | Gram stain | A | GMSA | B | GMSB | C | GMSC | D | GMSD |
| 6.77 | Bacterial cultures | A | BACA | B | BACB | C | BACC | D | BACD |
| 6.78 | Paediatric length-based (Broselow) tape | A | BSLA | B | BSLB | C | BSLC | D | BSLD |
| 6.79 | Otoscope | A | OTOA | B | OTOB | C | OTOC | D | OTOD |
| 6.80 | Ophthalmoscope | A | OPHA | B | OPHB | C | OPHC | D | OPHD |
| 6.81 | Gloves | A | GLOA | B | GLOB | C | GLOC | D | GLOD |
| 6.82 | Goggles | A | GOGA | B | GOGB | C | GOGC | D | GOGD |
| 6.83 | Sharps disposal | A | SHPA | B | SHPB | C | SHPC | D | SHPD |
| 6.84 | Biological waste disposal | A | BWDA | B | BWDB | C | BWDC | D | BWDD |
| 6.85 | Gowns | A | GOWA | B | GOWB | C | GOWC | D | GOWD |
| 6.86 | Post-exposure prophylaxis for HIV | A | PEPA | B | PEPB | C | PEPC | D | PEPD |

| Section 7. Trauma Care Medications | | | | | | | | | |
| --- | --- | --- | --- | --- | --- | --- | --- | --- | --- |
|  | Regarding Medications Important in the care of the injured: | A - Is this item present today?  YES = 1  NO = 0  Don’t know = 98  Missing = 99 | | B - If yes - Is a suitably trained individual able to use this item to care for the injured >90% of the time?  YES = 1  NO = 0  Don’t know = 98  Missing = 99 | | C - If Yes to B - When was this item last used to treat a patient?  Within the last week = 1  Within the last month = 2  Within the last 3 months = 3  Within the last 6 months = 4  Within the last year = 5  More than 1 year ago = 6  Never = 7  Not applicable = 97  Don’t know = 98  missing=99 | | D - If No to B - what is the reason? – tick all that apply  Lack of staff availability = 1  Lack of staff training = 2  Lack of staff motivation = 3  Lack of specialist services = 4  Lack of infrastructure = 5  Lack of equipment = 6  Lack of consumable material = 7  Lack of patient and family cooperation with care processes = 8  Lack of care processes such as policies and service organisation =9  Difficulties with payment for care = 10  Insufficient facility capacity – overcrowding =11  Other (specify)………… 12  Not applicable = 97  Don’t know = 98  Missing = 99 | |
| 7.1 | Bupivacaine (or equivalent) | A | BUPIA | B | BUPIB | C | BUPIC | D | BUPID |
| 7.2 | general anaesthetic (ether, halothane or equivalent) | A | GENAA | B | GENAB | C | GENAC | D | GENAD |
| 7.3 | ketamine | A | KETAA | B | KETAB | C | KETAC | D | KETAD |
| 7.4 | lidocaine (or equivalent) | A | LIDOA | B | LIDOB | C | LIDOC | D | LIDOD |
| 7.5 | nitrous oxide | A | NITOA | B | NITOB | C | NITOC | D | NITOD |
| 7.6 | thiopental (or equivalent) | A | THIOA | B | THIOB | C | THIOC | D | THIOD |
| 7.7 | diazepam (or equivalent) | A | DIAZA | B | DIAZB | C | DIAZC | D | DIAZD |
| 7.8 | atropine | A | ATROA | B | ATROB | C | ATROC | D | ATROD |
| 7.9 | morphine (or equivalent) | A | MORPA | B | MORPB | C | MORPC | D | MORPD |
| 7.10 | codeine (or equivalent) | A | CODEA | B | CODEB | C | CODEC | D | CODED |
| 7.11 | acetylsalicylic acid (aspirin) | A | ACSAA | B | ACSAB | C | ACSAC | D | ACSAD |
| 7.12 | ibuprofen (or equivalent) | A | IBUPA | B | IBUPB | C | IBUPC | D | IBUPD |
| 7.13 | paracetamol (acetaminophen) | A | PARAA | B | PARAB | C | PARAC | D | PARAD |
| 7.14 | dexamethasone, hydrocortisone (or other equivalent steroid) | A | DEXAA | B | DEXAB | C | DEXAC | D | DEXAD |
| 7.15 | epinephrine | A | EPINA | B | EPINB | C | EPINC | D | EPIND |
| 7.16 | naloxone | A | NALOA | B | NALOB | C | NALOC | D | NALOD |
| 7.17 | phenobarbital | A | PHEBA | B | PHEBB | C | PHEBC | D | PHEBD |
| 7.18 | phenytoin | A | PHEYA | B | PHEYB | C | PHEYC | D | PHEYD |
| 7.19 | magnesium sulphate | A | MGSOA | B | MGSOB | C | MGSOC | D | MGSOD |
| 7.20 | amoxycillin/ampicillin | A | AMOXA | B | AMOXB | C | AMOXC | D | AMOXD |
| 7.21 | amphotericin | A | AMPHA | B | AMPHB | C | AMPHC | D | AMPHD |
| 7.22 | benzylpenicillin | A | BENZA | B | BENZB | C | BENZC | D | BENZD |
| 7.23 | chloramphenicol | A | CHLOA | B | CHLOB | C | CHLOC | D | CHLOD |
| 7.24 | ciprofloxacin (or equivalent) | A | CIPRA | B | CIPRB | C | CIPRC | D | CIPRD |
| 7.25 | flucloxacillin (or equivalent) | A | FLUXA | B | FLUXB | C | FLUXC | D | FLUXD |
| 7.26 | fluconazole (or equivalent) | A | FLUZA | B | FLUZB | C | FLUZC | D | FLUZD |
| 7.27 | gentamicin (or equivalent) | A | GENTA | B | GENTB | C | GENTC | D | GENTD |
| 7.28 | metronidazole | A | METRA | B | METRB | C | METRC | D | METRD |
| 7.29 | sulfamethoxazole & trimethoprim (or equivalent) | A | TRIMA | B | TRIMB | C | TRIMC | D | TRIMD |
| 7.30 | heparin | A | HEPAA | B | HEPAB | C | HEPAC | D | HEPAD |
| 7.31 | warfarin (or equivalent) | A | WARFA | B | WARFB | C | WARFC | D | WARFD |
| 7.32 | sulfadiazine | A | SULFA | B | SULFB | C | SULFC | D | SULFD |
| 7.33 | antiseptics: chlorhexidine, ethanol, polyvidone or equivalent | A | ASEPA | B | ASEPB | C | ASEPC | D | ASEPD |
| 7.34 | disinfectants: chlorine base compound, chloroxylenol, glutaral or equivalent | A | DSINA | B | DSINB | C | DSINC | D | DSIND |
| 7.35 | furosemide (or equivalent) | A | FUROA | B | FUROB | C | FUROC | D | FUROD |
| 7.36 | aluminium hydroxide | A | ALOHA | B | ALOHB | C | ALOHC | D | ALOHD |
| 7.37 | cimetidine (or equivalent) | A | CIMEA | B | CIMEB | C | CIMEC | D | CIMED |
| 7.38 | magnesium hydroxide | A | MGOHA | B | MGOHB | C | MGOHC | D | MGOHD |
| 7.39 | insulin | A | INSUA | B | INSUB | C | INSUC | D | INSUD |
| 7.40 | alcuronium, suxamethonium or equivalent | A | RELXA | B | RELXB | C | RELXC | D | RELXD |
| 7.41 | neostigmine (or equivalent) | A | NEOSA | B | NEOSB | C | NEOSC | D | NEOSD |
| 7.42 | glucose solution (5%, 50%) | A | GLUSA | B | GLUSB | C | GLUSC | D | GLUSD |
| 7.43 | normal saline solution (0.9% isotonic) | A | NSALA | B | NSALB | C | NSALC | D | NSALD |
| 7.44 | Ringer's lactate solution (or equivalent) | A | RINGA | B | RINGB | C | RINGC | D | RINGD |
| 7.45 | potassium chloride solution | A | KCLSA | B | KCLSB | C | KCLSC | D | KCLSD |
