# Supplementary material for: Health system assessment for access to care after injury in low- or middle-income countries: A mixed methods study from Northern Malawi
Source: PLoS Med. 2024 Jan 22;21(1):e1004344. doi: 10.1371/journal.pmed.1004344 (PMC10843098; doi:10.1371/journal.pmed.1004344)
Supplement: S1 Table — (DOCX) [file pmed.1004344.s004.docx]

S1 Table – Mixed method integrated analysis detail of evidence grading for each study, for each of the three delays and identified barriers.

Legend - Barriers evidenced by 3 or more methods are included in the table. * denotes strength of evidence upgraded due to qualitative narrative description. FGD = Focus Group Discussion, HH = Household, HCW = Healthcare Worker, VA = Verbal Autopsy, GIS = Geographic Information System. Barriers for which a study methodology provided no evidence were categorised as silent. This silence could be a limitation of a method being unsuitable to detect evidence for a barrier, or suggestive that the barrier does not exist.

| **Delay 1 Overall** | | | | | | | | | | | | | | | | | | | | | | | | | | |
| --- | --- | --- | --- | --- | --- | --- | --- | --- | --- | --- | --- | --- | --- | --- | --- | --- | --- | --- | --- | --- | --- | --- | --- | --- | --- | --- |
| **Evidence from HH Survey** | **Evidence from Verbal Autopsy** | | | | | | **Evidence from FGDs** | | | | **Evidence from Photovoice** | | | | | | | **Evidence from Facility process mapping** | | | **Evidence from HCW survey** | | | | | |
| Overall Evidence for Delay 1 | | | | | | | | | | | | | | | | | | | | | | | | | | |
| 23.2% of those non fatally injured did not seek care following injury (142/611). This was mostly (93.7% 133/142) those with minor injuries (<30 days disability).  Evidence grade:  MODERATE | 65/120 (54.2%) VA deaths were assessed as potentially avoidable. Of these 20/65 (30.8%) delay 1 was implicated. In 11/65 (16.9%) cases it was judged the most important delay.  Evidence grade:  STRONG | | | | | | Description of 10 barriers acting to delay seeking care within the community. 5/10 (50.0%) raised by all 3 FGDs, 3/10 (30.0%) by 2 FGDs and 2/10 (20.0%) raised by 1 FGD.  Evidence grade:  STRONG | | | | Delay 1 was the most photographed of the 3 delays featuring in 12/21 (57.1%) photos. It also featured in an expressed desire to take a photo of a herbalist, but were not able to during the study.  Evidence grade:  STRONG | | | | | | | 8 delay 1 barriers featured frequently (6 or more / 11 facilities) in facility process maps. This was mostly for scenario 1.  Evidence grade:  STRONG | | | All healthcare workers thought at least some patients delayed Seeking care following injury. 52.2% (119/228) of Healthcare workers thought delays in seeking care following injury occurred in either about half, more than half or almost all cases. 40% though the first delay affected the Largest Number of injured people compared to delays 2 and 3. 19.3% thought delay 1 was the most important of the three delays in their population. Although most healthcare workers believed typical delays in seeking care were less than 4 hours 54.8% (125/228), 14.5% (33/228) reported these as being typically over 24 hours. Most healthcare workers, 86.4% (197/228) believed delaying seeking care to cause significant harm or worse  Evidence grade: STRONG | | | | | |
| **Delay 1 Barriers** | | | | | | | | | | | | | | | | | | | | | | | | | | |
| **Evidence from HH Survey** | | | | | **Evidence from Verbal Autopsy** | | | **Evidence from FGDs** | | | | | | | **Evidence from Community photovoice** | | | | | | **Evidence from Facility process mapping** | | | | | **Evidence from HCW survey** |
| COST - The financial costs associated with seeking care are too great | | | | | | | | | | | | | | | | | | | | | | | | | | |
| 11.3% (16/142) reported cost as a barrier to them seeking care. For 5.6% (8/142) this was the most important reason.  Evidence grade: STRONG | | | | | 2/20 (10.0%) delay 1 attributable cases of avoidable injury death had evidence of cost associated with causing delay to seeking care.  Evidence grade: MODERATE | | | This barrier was clearly expressed across all FGDs with a lack of money and the direct and indirect cost implications of seeking care preventing a decision to go.  Illustrative narrative  *“Sometimes the major problem is money for instance the father is there the and the mother is there but they have no money and … thinking about how he leaves the other family members .. at home without anything and wherever they take the patient …. if the money is not there this can also delay the idea of taking the patient to the hospital”*  Evidence grade: STRONG | | | | | | | 3 photos explained the barrier of cost as a potential barrier to seeking care.  Illustrative narrative  *“I took a picture of money, because most of the problems come when you do not have money. Whether you want to go to the hospital, even if you want to find quality care you need to have money, so this is why I took a picture of money”*  Evidence grade: STRONG | | | | | | Cost was frequently present (6/11 facilities) in process maps as a barrier to seeking care. Present in all scenarios though most commonly scenario 1.  Evidence grade: STRONG | | | | | Through the barrier scoring analysis, this was clearly the most important.  In the top 3 overall barrier analysis 38/228 (16.7%) reported this, the 6^th^ most cited barrier overall and 1^st^ amongst delay 1.  Evidence grade: STRONG |
| HEALTHCARE LITERACY- People don't understand about health and available healthcare | | | | | | | | | | | | | | | | | | | | | | | | | | |
| 60.6% (86/142) reported not seeking care because the injury was not serious enough. However most of these participants suffered minor injuries (disability <30 days).  In only 2.1% (3/142) of cases the injured person did not know healthcare was available.  Evidence grade: STRONG | | | | | This was the most commonly evidenced delay 1 barrier. 15/20 (75.0%) delay 1 attributable avoidable trauma deaths included narrative evidence.  Evidence grade: STRONG | | | This barrier was clearly described in detail by all three FGDs. Several domains were highlighted including the perceived severity, a community lack of awareness of post injury response, lack of recognition and perception of futility in more severe injuries.  Illustrative narrative  *“Sometimes we delay in taking somebody to the hospital because the patient is not showing any sign of fracture or he’s not bleeding at all, so we tend to think that he hasn’t been injured not knowing that the damage is internal.”*  Evidence grade: STRONG | | | | | | | Not evidenced  Evidence grade: SILENT | | | | | | This was frequently described by 8/11 facilities, most commonly for scenario 1 but across all scenarios.  Evidence grade: STRONG | | | | | This barrier was 4^th^ of 8 in the scoring analysis. 26/228 (11.4%) HCWs amongst the top 3 most important barriers, 2^nd^ of 8 delay 1 barriers and 9^th^ of 23 overall.  Evidence grade: MODERATE |
| CULTURAL NORMS - Normal cultural behaviours delay seeking care such as gender roles, family responsibilities and requiring someone else's permission to seek care | | | | | | | | | | | | | | | | | | | | | | | | | | |
| 15.5% (22/142) reported that the family member responsible for decisions about seeking care did not want the injured person to seek care. Although only considered the most important reason in 2.1% (3/142) of cases.  Evidence grade: STRONG | | | | | 10/20 (50.0%) delay 1 attributable avoidable trauma deaths had evidence from death narratives of cultural norms acting as a barrier to seeking care.  Evidence grade: STRONG | | | This was only described in FGD 3 (community leaders) in relation to male household heads traditionally making health seeking decisions for the family.  Illustrative narrative  *“At times there’s a tendency that no decision can be made at the household level when the head of the household is not around, so if there is an injury at home the other members wait until the head of household as arrived so that he can authorise the taking of injured person to the hospital and this can cause the delay.”*  Evidence grade: WEAK | | | | | | | Not evidenced  Evidence grade: SILENT | | | | | | This was frequently described (8/11 facilities), across all scenarios but only by 1 facility for scenario 2.  Evidence grade: STRONG | | | | | In scoring analysis this barrier was 2^nd^ out of 8.  It was chosen by 18/228 (7.9%) healthcare workers as amongst the top 3 most important health system barriers. 4^th^ of 8 in delay 1 and 14^th^ of 23 overall.  Evidence grade: STRONG |
| TRADITIONAL HEALERS- People prefer traditional healers | | | | | | | | | | | | | | | | | | | | | | | | | | |
| 10% (15/142) of those not seeking care following injury reported this as a reason. For 5% (7/142) this was the most important reason.  Evidence grade:  MODERATE | | | | | Only 1/20 (5.0%) Delay 1 attributable avoidable trauma deaths was a preference for traditional healers evident as a barrier to care.  Evidence grade:  WEAK | | | All 3 FGDs evidenced this barrier, often discussed in conjunction with other beliefs such as religious faith healing. Serious injuries and violent mechanisms were included in examples of traditional healing preference.  Illustrative narrative  *“Also some .. think that every accident takes place because of witchcraft, even if you fall from the roof or from a motorcycle or any other accidents like car accident they believe they happen because of witchcraft … so instead of taking the injured person to the hospital they will take the patient to a witch doctor believing that is where he will be treated forgetting that the witchdoctor cannot mend broken bones and cannot be in a position to rehydrate a dehydrated person so this person dies because of such beliefs and failure to get proper treatment at the hospital.”*  Evidence grade: STRONG | | | | | | | 2 photos were chosen by participants to highlight traditional healing beliefs as a barrier to seeking care.  Illustrative narrative  *“I took a picture of this banana plant because somebody had fractured his hand and instead of going to the hospital, he decided to use the roots of this banana plant. He thought he was getting better, but after 3 days the hand started swelling again and had some pus in it. All this was because he believed in herbs.”*  Evidence grade:  STRONG* | | | | | | This was a frequently reported by most facilities (8/11) primarily through scenario 1. Not mentioned by any facility for scenario 2 (assault and abdominal stab wound).  Evidence grade:  STRONG | | | | | From barrier scoring analysis it was the second least important (7^th^ of 8) delay 1 barrier.  20/228 (8.8%) HCWs placed this barrier within the top 3 most important overall. 3^rd^ of 8 in delay 1 and 12^th^ of 23 overall.  Evidence grade:  MODERATE |
| PERCEIVED PHYSICAL ACCESS - People perceive that care is too difficult to physically access | | | | | | | | | | | | | | | | | | | | | | | | | | |
| As a barrier to seeking care 19.0% (27/142) reported it being too difficult to get transport. For 13.4% this was the most important reason.  11.3% (16/142) reported the health facility was too far away, but only 1.4% (2/142) reported this as the most important.  Evidence grade: STRONG | | | | | Not evidenced  Evidence grade: SILENT | | | 2 FGDs (2&3) expressed this barrier. Highlighted were the physical distance between place of injury and care facility with  shorter distances lessening any financial travel burden. Challenging topography, including rivers, exacerbated by rainy season were expressed.  Illustrative narrative  *“sometimes the idea of taking the patient to the hospital is dismissed because of the distance of the hospital from where the patient is, for example there is an accident at Kasangamala and when people think about the distance from that place to Chilumba rural hospital they just give up and think it is just better to stay with the patient at home”*  Evidence grade: STRONG* | | | | | | | Not evidenced  Evidence grade: SILENT | | | | | | This barrier was reported frequently in facility maps (6/11), most commonly for scenario1 and only once for scenario 4 (motorcyclist hit by a taxi with leg fracture).  Evidence grade: STRONG | | | | | This barrier was 3^rd^ out of 8 most important using the scoring analysis. 7/228 (3.1%) healthcare workers included this amongst the top 3 overall most important barriers. 8^th^ of 8 in delay 1, 22^nd^ of 23 overall.  Evidence grade: MODERATE |
| COMMUNITY OR BYSTANDER ENGAGEMENT – Not enough is done by fellow citizens to support care seeking | | | | | | | | | | | | | | | | | | | | | | | | | | |
| 21.8% (31/142) reported the person or family had other priorities or responsibilities inhibiting them from seeking care.  Evidence grade: STRONG | | | | | Not evidenced  Evidence grade: SILENT | | | FGDs 2 and 3 expressed this barrier. Bystanders should be key care seeking enablers, who need courage to assist. Disagreements between bystanders can cause delays. They can be similarly preoccupied with their own affairs. Community leadership could legislate to improve care seeking following injury.  Illustrative narrative  *“The other thing that can delay a person to go to hospital is disagreements, sometimes when the person is severe injured instead of thinking to rushing with the patient to the hospital you delay because of disagreement.”*  Evidence grade: STRONG* | | | | | | | Three photos were describing the need for more community and bystander engagement. Focused on community preoccupation with other things to help their neighbour and the need for local legislation to support care seeking.  Illustrative narrative  *“I took this picture to show how people prioritize their own work over assisting those who are injured… In this picture I want you to see people peeling cassava and this shows people are busy with their own work and they don’t want to stop whatever they are doing. For example, when I got injured it was at night, and in the morning when I told my neighbours that I had an injury they told me that they will finish their work first and then take me to the hospital.”*  Evidence grade: STRONG | | | | | | Prioritise apportioning blame and liability for costs as an aspect of lack of community engagement was mentioned by only 1/11 facility.  Evidence grade: WEAK | | | | | Not evidenced  Evidence grade: SILENT |
| PERCEIVED CARE QUALITY - People perceive that available facility care is poor quality | | | | | | | | | | | | | | | | | | | | | | | | | | |
| 5% (7/142) reported that the health facility would not provide effective treatment, this was the most important reason for 4.2% (6/142). 3.5% (5/142) reported believing the health facility would not treat them with respect, for whom 1.2% (2/142) this was the most important reason for not seeking care. Although no one reported that the health facility would not communicate well with them. 2.1% (3/142) believed there was no clinician available at the facility. 1 participant reported negative previous facility experience and healthcare workers not responding in the right manner as reasons for not seeking care.  Evidence grade: MODERATE | | | | | Not evidenced  Evidence grade: SILENT | | | Described by all FGD groups driven by past experiences from self or others. Particularly non-technical aspects of care quality and structural inputs to care such as physical resources.  Illustrative narrative  *“sometimes we fail to go to the hospital in time because we have seen the doctors not paying attention to injured people they don’t welcome them when coming to hospital so you fail to take the injured person to hospital because you think of how you saw them treating others with same problems then you just think of staying at home or delay in bringing the injured person to the hospital”*  Evidence grade: STRONG | | | | | | | One photo and description touched on care quality perceptions amongst other barriers, framed as an encouragement for others to seek care when injured in the hope of good outcomes.  Illustrative narrative  *“I took a picture of that lady, because when I was asking her, she told me she was alive today because she was able to get care in time. This is an encouragement to those of us that get injured to go to the hospital the moment you are injured without hesitation.”*  Evidence grade: MODERATE* | | | | | | This was a frequently reported barrier (6/11 facilities), most commonly reported for scenario 1.  Evidence grade: STRONG | | | | | Ranked as 5^th^ out of 8 most important delay 1 barrier in scoring analysis.  12/228 (5.3%) including it within the top 3 overall barriers. 6^th^ of 8 within delay 1 and 18^th^ of 23 overall.  Evidence grade: MODERATE |
| DELAYED DISCOVERY - There are delays in discovering injured people, including because of intoxication | | | | | | | | | | | | | | | | | | | | | | | | | | |
| 1.4% (2/142) of cases the injured person reported being alone with no one to assist for which one case this was the most important reason.  Evidence grade: WEAK | | | | | 5/20 (25.0%) delay 1 attributable avoidable deaths had evidence of delayed discovery driving delay to seeking care.  Evidence grade: STRONG | | | Only described by one FGD and primarily in relation to a child’s injury not being disclosed.  Illustrative narrative  *“If I am unaware that my child is injured from where they were playing because they didn’t report to me, it can delay the child to go to hospital”*  Evidence grade: WEAK | | | | | | | Not evidenced  Evidence grade: SILENT | | | | | | This was only sometimes described, with 3/11 facilities reporting, and only for scenarios 1 and 3.  Evidence grade: MODERATE | | | | | This barrier was 6^th^ out of 8 in the scoring analysis.  10/228 (4.4%) healthcare workers reported it amongst the top 3 most important barriers overall. 7^th^ of 8 for delay 1 and 19^th^ of 23 overall.  Evidence grade: WEAK |
| RELIGIOUS OR OTHER BELIEFS – believing that seeking formal healthcare is itself wrong | | | | | | | | | | | | | | | | | | | | | | | | | | |
| 3.5% (5/142) reported the injured person did not believe that is was right to seek care following an injury. This was the most important reason for 2.1% (3/142)  Evidence grade: WEAK | | | | | Not evidenced.  Evidence grade: SILENT | | | FGD1 identified belief in healing through prayer and the church as distinct from traditional healing beliefs.  Illustrative narrative  *“some … injured will not go to the hospital because they believe that the church will heal them. They just to stay at home praying and believing that they’ll get healed.”*  Evidence grade: WEAK | | | | | | | 2 photos focused on other religious beliefs and preferring seeking healing through prayer.  Illustrative narrative  *“I wanted to show .. that some people because of their beliefs don’t like going to the hospital. They choose to be prayed for... They belief that it is God that has allowed the person to have an accident and it is God that can remove the problem the person is having.”*  Evidence grade: MODERATE | | | | | | Religious beliefs that seeking formal healthcare is wrong featured frequently, in 6/11 facility process maps, with all scenarios implicated at least once.  Evidence grade: STRONG | | | | | Not evidenced.  Evidence grade: SILENT |
| FEAR OR LACKING COURAGE – irrational incapacitation or rational concern of consequences such as being accused | | | | | | | | | | | | | | | | | | | | | | | | | | |
| 1.4% (2/142) injured non care seekers reported fear as a reason. 0.7% (1/142) reported this as the most important.  Evidence grade: WEAK | | | | | Not evidenced  Evidence grade: SILENT | | | FGD 2 and 3 expressed this barrier, both in relation to a parent becoming incapacitated when a child is injured, and courage from bystanders to assist a fellow citizen.  Illustrative narrative  *“Sometimes accidents happen and people lack courage to carry that patient to the hospital”*  Evidence grade: MODERATE | | | | | | | Not evidenced  Evidence grade: SILENT | | | | | | People fear the consequences of helping an injured person, e.g. being accused of causing the injury was evident and sometimes reported – 5/11 facilities – primarily related to scenario 2 – assault with one facility also relating this to scenario 4 – road traffic collision.  Evidence grade: MODERATE | | | | | This barrier affected the fewest number and caused the least delay according to the scoring analysis. However 14/228 (6.1%) still chose it as amongst the top 3 most important health system barriers overall. 17 of 23.  Evidence grade: WEAK |
| **Delay 2 Overall** | | | | | | | | | | | | | | | | | | | | | | | | | | |
| **Evidence from HH Survey** | | | **Evidence from Verbal Autopsy** | | | | | | **Evidence from FGDs** | | | **Evidence from Community photovoice** | | | | **Evidence from Facility process mapping** | | | | **Evidence from HCW survey** | | | | **GIS** | | |
| Overall Evidence for Delay 2 | | | | | | | | | | | | | | | | | | | | | | | | | | |
| 29.6% (138/467) of those seeking care attended a second place of care subsequently.  61.0% (285/467) reached care (1^st^ facility) within 1 hour of deciding to seek care. 88.8% (415/467) reached care within 2 hours.  Evidence Grade: MODERATE | | | 65/120 (54.2%) VA deaths were assessed as potentially avoidable. Delay 2 was implicated in 40/65 (61.5%) avoidable trauma deaths. In 15/65 (23.1%) cases it was judged the most important delay.  Evidence Grade:  STRONG | | | | | | 4 delay 2 barriers were evidenced, 1 by all 3 FGDs, 3 by 2 FGDs.  Evidence Grade:  STRONG | | | 10/21 (47.6%) photos provided evidence of delay 2 barriers.  Evidence Grade:  STRONG | | | | Multiple barriers frequently described within delay 2. 5 barriers were reported frequently (6 or more facilities) and 4 were reported sometimes (3 to 5 facilities).  Evidence Grade:  STRONG | | | | 43.9% (100/228) believed delay 2 affected the largest number of injured people. Similarly, overall 44.2% (101/228) believed delay 2 caused the most delay to those injured people affected.  Evidence Grade:  STRONG | | | | For proportion of population reaching facilities within timelines as follows:  Primary any – 94% <1 hour  Chilumba rural hospital – 64.9% <1 hour  Secondary any – 28.2% <1hour, 50.9%1 to 2 hours  Karonga District Hospital (main government secondary hospital) – 0%<1 hour, 20% <2 hours, 70% 2 to 4 hours  Mzuzu Central Hospital (government tertiary hospital) – 27.6% 2 to 4 hours, 64.3% 4 to 6 hours  Evidence Grade:  MODERATE | | |
| **Delay 2 Barriers** | | | | | | | | | | | | | | | | | | | | | | | | | | |
| **Evidence from Verbal Autopsy** | | | | | | **Evidence from FGDs** | | | | | | | | | | | **Evidence from Community photovoice** | | | | | | **Evidence from Facility process mapping** | | | **Evidence from HCW survey** |
| TRANSPORT - There is a lack of timely affordable emergency transport (formal or informal) | | | | | | | | | | | | | | | | | | | | | | | | | | |
| Barrier evident in 13/40 (32.5%) delay 2 related avoidable deaths.  Evidence Grade: STRONG | | | | | | This barrier was repeatedly emphasised by all discussion groups both in terms of whether or not a means of transport could be readily located or obtained, and the cost of such transport.  Illustrative narrative  *“what will determine the type of transport … being rich in the pocket … you can have a wheelbarrow …to take the patient to the hospital if it’s closer to the incident place and depending on the weight of the patient, ..you can carry the patient on the back .. according to severity of the injury .. and if it’s closer to the hospital. And maybe the good Samaritans .. can come in to help take the patient to the hospital since … and …the patient can be taken to the hospital using the bicycle according to the condition of the patient..”*  *“if somebody carries you in his vehicle upon reaching the hospital (he) will demand payment straight away“*  Evidence Grade: STRONG | | | | | | | | | | | 5/21 (23,8%) photos highlighted transport and particularly its cost as a barrier to reaching care  Illustrative narrative  *“That it is an oxcart which we use most of the times because it is difficult for us to … look for better transport… When I got injured, .. I used that of a neighbour, and though I was charged, I was told to pay when I am ready…It took us quite a long time before we got to the hospital at jetty (Chilumba rural hospital) I think it took us 3 hours …as you know it is sandy here and the animals don’t walk very fast.”*  Evidence Grade: STRONG | | | | | | All 11 facilities reported this barrier in most scenarios.  Evidence Grade: STRONG | | | In scoring analysis this was the most important delay 2 barrier.  68/228 (29.8%) reported this amongst the top 3 overall barriers. 1st of 6 for delay 2, 3rd of 23 overall.  Evidence Grade: STRONG |
| ROADS - There is a lack of reliable uncongested roads with priority for emergency vehicles | | | | | | | | | | | | | | | | | | | | | | | | | | |
| This barrier was evident in 11/40 (27.5%) delay 2 avoidable deaths.  Evidence Grade: STRONG | | | | | | This was discussed extensively in FGD1 and also in FGD3. This focused on the poor unreliable condition and a marked deterioration during rainy season.  Illustrative narrative  *“we are referring to houses that are built in mountainous areas and because of the rains roads become bad and accessibility difficult and so it becomes difficult for any vehicle to come and pick this patient”*  Evidence Grade: STRONG* | | | | | | | | | | | 6/21 (28.6%) photos focused on this barrier  Illustrative narrative  *“This is an example of a bad road… a patient has come from very remote areas, coming to Hara clinic, and if it found that the patient has to go to Chilumba rural hospital they will have to use this bad road … The dark spots that you are seeing are the potholes on the road, there are very many of them making it very difficult for a patient to reach the hospital because the road is very bumpy…”*  Evidence Grade: STRONG | | | | | | This was frequently reported in 6/11 facilities across all 4 scenarios.  Evidence Grade: STRONG | | | This was clearly the least important delay 2 barrier according to the scoring analysis.  Only 8/228 (3.5%) rated this amongst the top 3 most important overall. The least for a delay 2 barrier. 21^st^ of 23 overall.  Evidence Grade: WEAK |
| DISTANCE - There is a large physical distance from place of injury to an appropriate healthcare facility | | | | | | | | | | | | | | | | | | | | | | | | | | |
| Evident in 17/40 (42.5%) delay 2 related avoidable deaths.  Evidence Grade:  STRONG | | | | | | This factor was mentioned as both restricting transport options and providing difficulties of itself by FGD 2 and 3.  Illustrative narrative  *“Sometimes where the accident took place is very far .. such that transport like bicycle, wheelbarrow or oxcart can’t reach .. making it hard to reach the hospital in time”*  Evidence Grade:  STRONG* | | | | | | | | | | | Not evidenced  Evidence Grade: SILENT | | | | | | Frequently reported (8/11 facilities) and across the 4 scenarios.  Evidence Grade: STRONG | | | Second most important delay 2 barrier in scoring analysis.  31/228 (13.6%) reported this amongst the top 3 overall barriers. 2nd of 6 for delay 2, 7th of 23 overall.  Evidence Grade: STRONG |
| PREHOSPITAL CARE - There is a lack of timely available prehospital emergency care (formal or informal/bystander) | | | | | | | | | | | | | | | | | | | | | | | | | | |
| Evident in 37/40 (92.5%) delay 2 related avoidable deaths.  Evidence Grade: STRONG | | | | | | Not evidenced  Evidence Grade: SILENT | | | | | | | | | | | Not evidenced  Evidence Grade: SILENT | | | | | | Rarely explicitly discussed, only 2/11 facilities raised this barrier specifically.  Evidence Grade: WEAK | | | In barrier scoring this was 3rd of 6 in importance.  25/228 (11.0%) reported this amongst the top 3 overall barriers. 4^th^ of 6 for delay 2, 10^th^ of 23 overall.  Evidence Grade: MODERATE |
| COMMUNICATION - There is a lack of accessible emergency assistance communication mechanism (e.g. emergency call centre) | | | | | | | | | | | | | | | | | | | | | | | | | | |
| Evident in 37/40 (92.5%) delay 2 related avoidable deaths.  Evidence Grade: STRONG | | | | | | Not evidenced  Evidence Grade: SILENT | | | | | | | | | | | Not evidenced  Evidence Grade: SILENT | | | | | | Rarely explicitly discussed, only 1/11 facilities raised this barrier specifically.  Evidence Grade: WEAK | | | In barrier scoring this was 4^th^ of 6 in importance.  29/228 (12.7%) reported this amongst the top 3 overall barriers. 3rd of 6 for delay 2, 8^th^ of 23 overall.  Evidence Grade: MODERATE |
| COORDINATION - There is a lack of emergency care service coordination, including bypassing unsuitable facilities | | | | | | | | | | | | | | | | | | | | | | | | | | |
| Evident in 17/40 (42.5%) delay 2 related avoidable deaths.  Evidence Grade:  STRONG | | | | | | Not evidenced  Evidence Grade:  SILENT | | | | | | | | | | | Not evidenced  Evidence Grade:  SILENT | | | | | | Rarely explicitly discussed, only 2/11 facilities raised this barrier specifically.  Evidence Grade:  WEAK | | | In barrier scoring this was 5th of 6 in importance.  15/228 (6.6%) reported this amongst the top 3 overall barriers. 5th of 6 for delay 2, 16^th^ of 23 overall.  Evidence Grade:  WEAK |
| **Delay 3 overall** | | | | | | | | | | | | | | | | | | | | | | | | | | |
| **Evidence from HH Survey** | | | | **Evidence from Verbal Autopsy** | | | **Evidence from FGDs** | | | **Evidence from Community photovoice** | | | | **Evidence from Facility process mapping** | | | | | **Evidence from HCW survey** | | | | **Facility Assessment** | | | **Clinical Vignettes** |
| Overall Evidence for Delay 3 | | | | | | | | | | | | | | | | | | | | | | | | | | |
| The majority, 70.7% (330/467) received care within 1 hour of arriving at a health facility and 90.4% (422/467) within 2 hours. Over three quarters, 77.3% (361/467), were either satisfied or very satisfied with the quality of care received.  Evidence Grade:  WEAK | | | | 65/120 (54.2%) VA deaths were assessed as potentially avoidable. 49/60 (81.7%) avoidable trauma deaths were attributed to delay 3. Delay 3 was the most important in 39/65 (60.0%).  Evidence Grade:  STRONG | | | 9 delay 3 barriers were evidenced. 3 by all FGDs, 5 by 2 FGDs and 1 by only 1 FGD.  Evidence Grade:  STRONG | | | 7/21 (30.0%) photos captured barriers related to delay 3.  Evidence Grade:  STRONG | | | | 4 barriers were reported frequently (6/11 or more facilities) 4 barriers described sometimes (3 to 5/11 facilities)  Evidence Grade:  STRONG | | | | | The majority of participants, 71.5% (163/228) believed “few” or no patients experienced delays in receiving care at a facility. Similarly, the majority, 57.0% (130/228) believed that typical delays to receiving care were less than 1 hour. Most participants, 67.1% (153/228), believed that those who have experienced delays in receiving care were likely to have suffered “significant harm” or “very significant harm”.  Delay 3 was considered the easiest delay to change by 57% of HCWs and the most important by 35.1%.  Evidence Grade: MODERATE | | | | No facility had 100% of the physical resources deemed essential by the World Health Organisation Essential Trauma Care checklist at any level of facility. Primary facilities ranged from 36% to 86% of essential resources required at a basic facility whilst secondary facilities had from 79% to 86% and the tertiary facility 93%. Primary facilities ranged from 30% to 73% of essential resources required at a General Practitioner Hospital facility whilst secondary facilities had from 81% to 84% and the tertiary facility 92%. Primary facilities ranged from 20% to 52% of essential resources required at a specialist facility whilst secondary facilities had from 72% and the tertiary facility 89%.  Evidence Grade:  STRONG | | | Deficiencies in best practice care were evidence across all scenarios of hypothetical injured patients.  Evidence Grade:  STRONG |
| **Delay 3 Barriers** | | | | | | | | | | | | | | | | | | | | | | | | | | |
| **Evidence from Verbal Autopsy** | | **Evidence from FGDs** | | | | | **Evidence from Community photovoice** | | | | | | **Evidence from Facility process mapping** | | | | | | **Evidence from HCW survey** | | | **Facility Assessment** | | | **Clinical Vignettes** | |
| PHYSICAL RESOURCES  to There is a lack of reliably available necessary physical resources (e.g. infrastructure, equipment and consumable material) | | | | | | | | | | | | | | | | | | | | | | | | | | |
| 42/49 (85.7%) delay 3 deaths had this barrier implicated.  Evidence Grade:  STRONG | | This was raised across all the FGDs in relation to medication availability, physical equipment availability and reliable electricity.  Illustrative narrative  *“sometimes you get to the hospital and doctors are there but you are told that your patient is dehydrated …the hospital is not able to provide you are told to go and buy somewhere … I think we can call it lack of equipment or resources.”*  Evidence Grade:  STRONG | | | | | 2 photos incorporated the barrier of lack of physical resources.  Illustrative narrative  *“sometimes I can fail to get care because of lack of equipment, and they can tell me everything went out of stock two days ago and I may die because of lack of equipment and yet I am already at the hospital.“*  Evidence Grade: STRONG* | | | | | | This barrier was frequently reported (9/11 facilities) within facility process mapping and distributed across the scenarios.  Evidence Grade:  STRONG | | | | | | In barrier scoring analysis this was clearly the most important, affecting both the most number and causing the greatest delay.  Similarly across all delays it was most commonly cited amongst the top 3 barriers (95/228, 41.7%), 1^st^ of 23.  Evidence Grade:  STRONG | | | For resources required to manage the sentinel injuries the tertiary facility had between 93 to 100% of resources required to manage the four injury scenarios. For the secondary facilities this ranged from 76 to 91% and for 33 to 78% for primary facilities. No primary facility had more than 60% of the required resources to manage all four sentinel injuries.  Evidence Grade:  STRONG | | | Unavailability of the required resource was the almost universal reason given for being unable to do something that ideally should be done in the vignette scenarios.  Evidence Grade:  STRONG | |
| STAFF - In regards to staffing, there is a lack of reliably available, suitably trained and motivated clinical staff | | | | | | | | | | | | | | | | | | | | | | | | | | |
| 34/49 (69.4%) avoidable deaths evidenced this barrier.  Evidence Grade:  STRONG | | All 3 FGDs raised this barrier. Multiple aspects of this barrier were included. Availability of staff including accessibility outside routine working hours. And non-technical aspects of care through staff motivation and staff attitude.  Illustrative narrative  *“what of the times people down Karonga … delay to receive care whilst at the hospital … doctors are found at the hospital they just come and look at the patient and they go away … otherwise such people … treated in good time … will .. not be dying when they are already at the hospital.”*  Evidence Grade:  STRONG | | | | | Only 1 photo described this barrier.  Illustrative narrative  *“the picture …is a hospital ... I took this picture because I went to the hospital when I got injured and the doctors did not help me.”*  Evidence Grade:  MODERATE* | | | | | | This barrier was frequently described by 7/11 facilities across all scenarios.  Evidence Grade:  STRONG | | | | | | In scoring analysis this was the second most important delay 3 barrier.  89/228 (39.0%) rated this amongst the top 3 most important overall. 2^nd^ of 9 delay 3 barriers and 2^nd^ of 23 overall.  Evidence Grade:  STRONG | | | No doctors work in primary facilities. Non-physician clinicians provide most care at primary and secondary facilities. Only one primary facility had provider with post qualification training in injury. This applied to only 1 doctor in secondary facilities and 22 non physician clinicians and 4 specialist doctors in tertiary care.  Evidence Grade: STRONG | | | The mean score for all providers across all vignette scenarios was only 49.4% (i.e. half of important care elements were not mentioned). Compared to doctors the score was significantly lower for Clinical Officers and Medical Assistants who provide all primary and most secondary care.  Evidence Grade:  STRONG | |
| SPECIALISTS - There is a lack of reliable timely access to specialist injury care services | | | | | | | | | | | | | | | | | | | | | | | | | | |
| 19/49 (38.8%) delay 3 avoidable deaths evidenced this barrier.  Evidence Grade:  STRONG | | This barrier was highlighted by all FGDs. Local inability to managing certain injuries contributed to avoidable morbidity and mortality. Specialists were felt to motivate better care amongst junior colleagues.  Illustrative narrative  *“the other thing that delays us to receive care .. depends on the doctors you have found at the hospital. Let’s take here in Chilumba, one may bleed internally due to accident but they can’t check inside quickly because there is no expert doctor they will keep you may be after two to three days is when they refer you to Karonga while the person is damaged internally and .. even reach there already dead”*  Evidence Grade:  STRONG | | | | | Only 1 photo covered this barrier, related to lack of local expertise for injury care.  Illustrative narrative  *“Sometimes I am injured, and I get to the hospital .., but I am not able to get the needed care because the doctor that I find there has no expertise on my problem, or the expert is not around. So, this can also cause me to fail to get the needed care.”*  Evidence Grade: MODERATE* | | | | | | Only 1 facility described this barrier and only in relation to scenario 2.  Evidence Grade: WEAK | | | | | | In scoring analysis this was the third most important barrier in delay 3.  43/228 (18.9%) rated this amongst the top 3 most important overall. 4^th^ of 9 in delay 3 and 5^th^ of 23 overall.  Evidence Grade:  STRONG | | | No specialist medical doctors were available in primary or secondary care. Although specialist orthopaedic officers provided orthopaedic care in secondary facilities.  Evidence Grade:  STRONG | | | Most participants in vignette exercise, i.e. available practitioners for caring for the injured were non doctors, and those doctors available to participate were generalists.  Evidence Grade:  STRONG | |
| INTERFACILITY TRANSFER - Lack of available means to safely and quickly transfer injured patients on to a more specialist facility | | | | | | | | | | | | | | | | | | | | | | | | | | |
| 18/49 (36.7%) avoidable delay 3 related deaths featured this barrier.  Evidence Grade:  STRONG | | Barrier describe by FGD 1 and 3 noting limited formal ambulance interfacility transfer necessitating alternative private transport or financing cost of ambulance fuel.  Illustrative narrative  “let me share my own experience … there were a number of us sustained injuries, we were … in private cars to Chilumba rural hospital but from there, there was only one ambulance which carried two people to Karonga district hospital …. because they was no other cars to take us to Karonga district hospital consequently some accident victims died before getting any care.”  Evidence Grade: STRONG* | | | | | 1 photovoice photo described this barrier.  Illustrative narrative  *“when they were wanting to refer us to Karonga district hospital, the ambulance was not there. I remember somebody offered his vehicle and because there were many of us there was no way we could sit up properly, but we were packed like bags of fertilizer. On the way to Karonga, we had a lot of problems and because of the pain people will throw their legs anywhere and in the process hurting others and some people died on the way.”*  Evidence Grade:  STRONG* | | | | | | Frequently described by 8/11 facilities including all 4 scenarios.  Evidence Grade:  STRONG | | | | | | In scoring analysis this was 5^th^ out of 9 within delay 3.  49/228 (21.5%) rated this amongst the top 3 most important overall. 3^rd^ of 9 in delay 3 and 4^th^ of 23 overall.  Evidence Grade:  STRONG | | | 8 facilities reported an ambulance or other vehicle for emergency transportation operating from the facility. 2/3 facilities without this reported they had access to an emergency vehicle stationed elsewhere. Fuel was reported as available on the day of visit in all 8 facilities. 2 facilities reported the emergency vehicle as having equipment to treat injured persons. With 3 facilities reporting ambulance staff as able to provide some form of care to injured patients. The main role of these vehicles was the transfer of patients from the facility to another more specialist facility. This varied in frequency of use to most days for the government secondary facility to less than every year for a government primary facility.  Evidence Grade:  MODERATE | | | Although arranging transfer was part of best practice scoring, the vignettes weren’t able to judge whether this process functioned as a barrier.  Evidence Grade:  SILENT | |
| QUALITY PROCESSES- There is a lack of good quality, consistent, structured, clinical priority driven injury care processes | | | | | | | | | | | | | | | | | | | | | | | | | | |
| 35/49 (71.4%) delay 3 avoidable deaths suggested this barrier.  Evidence Grade:  STRONG | | FGD 1 and 3 both described this barrier in relation to inherent delays with out of hours care arrangements and  a need to prioritise urgent injured cases based on clinical need.  Illustrative narrative  *“when you are on queue … At the hospital, if the injured person is on row number 5 the doctor should first attend the injured person instead of attending the whole queue and then continue attending other people”*  Evidence Grade: STRONG* | | | | | 1 image touched on this barrier amongst others.  Illustrative narrative  *“I took a picture of that lady.., she told me she was alive today because she was able to get care in time. This is an encouragement to those of us that get injured to go to the hospital the moment you are injured without hesitation.”*  Evidence Grade:  MODERATE* | | | | | | This was sometimes found in process maps (3/11) across all scenarios.  Evidence Grade:  MODERATE | | | | | | This was 4^th^ out of 9 in scoring analysis.  19/228 (8.3%) rated this amongst the top 3 most important barriers overall. 6^th^ of 9 for delay 3, 13^th^ of 23 overall.  Evidence Grade:  MODERATE | | | No facility reported maintaining a specific trauma registry of cases. Only 1 facility reported any policy for the management of injured persons. Only 1 facility reported designating a team for managing injured persons (Karonga District Hospital). This was for multi-casualty scenarios rather than individual injured cases. Three facilities reported requiring some staff to undergo specific training in the care of the injured. No facilities reported conducting a review of injury related deaths.  Evidence Grade:  STRONG | | | No individual scored 100% in any scenario. In scenario 1 feeling for tracheal deviation was done by 7.1%. In scenario 2 no participant mentioned tranexamic acid. In scenario 3. Only 7.1% ensured a normal blood pressure. In scenario 4 assessing lower limb distal neurology was mentioned by 2.4%.  Evidence Grade:  STRONG | |
| ALLEGED CORRUPTION - Need for unauthorised payments or gifts to healthcare staff to receive best available treatment. (e.g. corruption) | | | | | | | | | | | | | | | | | | | | | | | | | | |
| Not evidenced  Evidence Grade: SILENT | | Raised by FGDs 1 and 2 relating to a requirement to provide additional payments to receive care promptly, although not a guarantee. Also  being well known to facility staff could facilitate prompt treatment.  Illustrative narrative  *“Sometimes corruption comes into play for example if you have nothing in your hands the doctors will just look at your patient without even paying attention … but the moment you give them some those … working on your patient provide all the care that is required..”*  Evidence Grade: STRONG* | | | | | 2 photovoice photos discussed this barrier.  Illustrative narrative  *“ my junior sister’s child had an injury … and the child had lost a lot of blood. She needed a blood transfusion …the doctors told us that the form … which showed that she was supposed to receive blood had gone missing.… a junior sister who was staying there….met the doctor and gave him a little something… and just before an hour we were told that the form was found and immediately … they got blood from the child’s father and gave it to the child.”*  Evidence Grade: STRONG* | | | | | | Not evidenced  Evidence Grade: SILENT | | | | | | 9^th^ out of 9 barriers in scoring analysis.  8/228 (3.5%) HCWs rated this amongst the top 3 overall barriers. 8^th^ out of 9 for delay 3 and 20/23 (87.0%) overall.  Evidence Grade: WEAK | | | Not able to assess  Evidence Grade: SILENT | | | Not able to assess  Evidence Grade: SILENT | |
| POLICE PROCESSES – Perceived or actual police functions affect care access | | | | | | | | | | | | | | | | | | | | | | | | | | |
| Police processes delayed care seeking according to VA analysis for 1/20 (5.0%) delay 1 attributed avoidable death.  There is controversy over where this barrier sits within the 3 delays.  Evidence Grade: WEAK | | FGD 3 described police processes as a barrier primarily acting at the level of facility, being redirected to the police station on reaching care.  Injured persons can be required to obtain a police report prior to receiving care.  This was dependent on the mechanism of injury involving a third party and enforced by hospital staff.  Illustrative narrative  *“Sometimes injuries are caused by other people … when you get to the hospital you’re told that you need to go to the police station so that you can write a statement before the hospital can assist you. When you get to the police station you will find that there is still a big queue and that means you have to wait and you time comes.”*  Evidence Grade: MODERATE* | | | | | Not evidenced  Evidence Grade: SILENT | | | | | | Within facility process mapping delays seeking care were described due to the need (perceived or actual) for a police process (e.g. report).  8/11 facilities described this in relation to assault and 4/11 in relation to a road traffic collision. Although this was conceived as within delay 1.  Evidence Grade: STRONG | | | | | | 1 HCW described this barrier using other free text. It was framed as delay 3.  Evidence Grade: WEAK | | | Not able to assess  Evidence Grade: SILENT | | | Not able to assess  Evidence Grade: SILENT | |
| PAYMENT - Difficulties with timely payment for care | | | | | | | | | | | | | | | | | | | | | | | | | | |
| 1/49 (2.0%) delay 3 avoidable deaths revealed evidence of this barrier.  Evidence Grade: WEAK | | FGD 1 and 3 raised this barrier noting direct care payments required for private, including faith based, providers. Related was that patients perceived to be wealthy could receive prompt better care.  Illustrative narrative  *“for mission hospitals for you to be assisted in good time you ought to have … availability of money.”*  Evidence Grade: MODERATE | | | | | 2 photos described this barrier  Illustrative narrative  *“this animal can be used ..as cash … because at times the money can be used to settle medical bills.”*  Evidence Grade: MODERATE | | | | | | Only 1/11 facility incorporated this barrier into its process map.  Evidence Grade: WEAK | | | | | | This barrier was 8^th^ out of 9 in scoring analysis.  4/228 (1.8%) rated this amongst the top 3 overall. The least of all barriers in all delays.  Evidence Grade: WEAK | | | Not able to assess  Evidence Grade: SILENT | | | Not able to assess  Evidence Grade: SILENT | |
| PATIENT COOPERATION - There is a lack of patient and family cooperation with care processes | | | | | | | | | | | | | | | | | | | | | | | | | | |
| Not evidenced  Evidence Grade: SILENT | | This barrier was clearly described in FGD 1 and 3. It included providing accurate information about the patient. Compliance with the prescribed treatment and guardians advocating for patients congniscent of clinician’s other patient responsibilities.  Illustrative narrative  *“the injured person must provide necessary information in good time to the doctor .. to receive the treatment in time, some people when they went to the hospital they fail to provide necessary information … the way they got injured … so how will the doctor know?”*  Evidence Grade: MODERATE | | | | | Not evidenced  Evidence Grade: SILENT | | | | | | Only one scenario in one facility suggested this barrier.  Evidence Grade: WEAK | | | | | | This was 7^th^ of 9 in importance in scoring analysis.  15/228 (6.6%) rated this amongst the top 3 most important overall. 7^th^ out of 9 delay 3 barriers, 15^th^ of 23.  Evidence Grade: MODERATE | | | Not able to assess  Evidence Grade: SILENT | | | Not able to assess  Evidence Grade: SILENT | |
| CAPACITY - In regards to patient demand, there is insufficient facility capacity to meet patient demand (e.g. overcrowding) | | | | | | | | | | | | | | | | | | | | | | | | | | |
| Not evidenced  Evidence Grade: SILENT | | Not evidenced  Evidence Grade: SILENT | | | | | Not evidenced  Evidence Grade: SILENT | | | | | | Sometimes featured, 4/11 process maps.  Evidence Grade: MODERATE | | | | | | In scoring analysis this was 6^th^ out of 9 barriers.  20/228 (8.8%) rated this amongst the top 3 overall barriers. 5^th^ out of 9 for delay 3, 11/23 overall.  Evidence Grade: MODERATE | | | Not able to assess  Evidence Grade: SILENT | | | Not able to assess  Evidence Grade: SILENT | |
