# Supplementary material for: Health system assessment for access to care after injury in low- or middle-income countries: A mixed methods study from Northern Malawi
Source: PLoS Med. 2024 Jan 22;21(1):e1004344. doi: 10.1371/journal.pmed.1004344 (PMC10843098; doi:10.1371/journal.pmed.1004344)
Supplement: S2 Table — (DOCX) [file pmed.1004344.s005.docx]

**S2 Table Detail of reflections on the compliance of study methods with the rapid assessment principles (authors’ judgement).**

Legend - Performance refers to authors’ experience, and potential refers to the scope for adaptation to improve compliance with a rapid assessment principle. These represent the authors’ reflections.

Well-aligned (green), partly well aligned (yellow), or poorly aligned (red).

|  | **Speed** | **Pragmatism** | **Cost-effectiveness.** | **Triangulation with Multiple data sources** |
| --- | --- | --- | --- | --- |
| **Household Survey** | | | | |
| Performance | Data collection mostly complete inside two months including research assistant training and piloting. | Local language competence required by research assistants. Health and Demographic Surveillance Site used as sampling frame. | Most costly method used due to human resources requires. | Integrated effectively although more detailed barrier focussed questions could have targeted delays 2 and 3. |
| Potential | More resource could speed up substantially. | Likely to remain onerous to collect data. | Will remain resource intensive in proportion to sample size needed. | Can be a valuable source of community quantitative data for triangulation |
| **Verbal Autopsy Analysis** | | | | |
| Performance | Secondary data analysis although may take time sorting through narratives. | Valuable source of "good enough" data to interpret meaning about the available health system for community trauma-related deaths. | Secondary analysis of database was low cost, requiring researcher time only. | Analysis is of data collected for another purpose.  Identification of specific phenomena or barriers may not be possible (e.g. perceptions).  Misses non-fatal burden. |
| Potential | As above | Unlikely to be available outside of resourced and funded academic research organisation. | If available is cost-effective | Will miss much of burden and limited insights to those extractable. |
| **Community Focus Group Discussion** | | | | |
| Performance | Data collection was rapid although translation and transcription were delayed in my study. | Purposive sampling requires cultural insight. To identify recently injured community members benefited from an embedded research partner. | Relatively inexpensive to collate. Small costs for participant refreshments, a venue if necessary, staff for training, conducting Focus Group Discussions and subsequent transcription. | Different Focus Group Discussion perspectives offered convergence of findings but also additional breadth. Integrated effectively with other studies. |
| Potential | Dedicated translation services would mitigate such delays. | Other pragmatic sampling strategies could be used instead. | Similar costs likely apply | Useful approach for confirming findings from other studies, focussed questions could compliment. |
| **Community Photovoice** | | | | |
| Performance | Data collection complete in one week with transcription taking two days dedicated work. | Ethical challenges include participant security and taking photos of individuals. Emancipatory value contested. Some physical resource logistics related to photography equipment and images need overcoming. | Relatively inexpensive to collate. Cameras can be costly, but we used low-cost devices.  Management of lost cameras needs to be considered. | Useful for deepening understanding, may not generate as many new ideas as Focus Group Discussions.  Might be focussed on where the participants can access. Use of the abstract was not as evident. |
| Likely Potential | Short data collection periods are common. More time could allow more reflection and photos. | Growth in smartphone access may ease logistics. | Low costs likely to remain achievable | Photo-elicitation of phenomena potentially deepening and enhancing exploration and understanding. |
| **Facility process mapping** | | | | |
| Performance | Each workshop took only a few hours with a similar time frame for map consolidation. | Pragmatic purposive staff sampling engaged a range of views. English language required with widespread electronic communication use enabling validation. | Minimal physical resource requirement and therefore cost. | Different healthcare worker perspectives encouraged.  This generated the greatest number of barrier ideas across all studies. |
| Likely Potential | Likely to remain a quick way to generate insight, determined by the number of workshops desired for a specific study. | Likely to remain highly practical, although validation may depend on electronic communication access. | Additional resources are unlikely to be necessary. May need translators if not in researcher's language. | Multiple perspectives are possible though caution over the role of hierarchical power dynamics in group workshop participation. |
| **Healthcare Worker survey** | | | | |
| Performance | The administered survey was relatively time-consuming. | Conducted in Chitumbuka needing fluent interviewers. Pragmatic sampling frame. Accessing staff limited to quiet facility times. | Two trained interviewers required. Use of electronic data capture minimised cost. | Additional barrier generation was minimal. But useful for quantitative value on perspectives. |
| Likely Potential | Self-administration could speed up, at likely cost of participation in number and breadth of participants. | Ability to conduct this remotely possible but challenging to capture the number and type of participants I did. | Self-administration could lower cost, but administration with interviewers likely necessary for similar breadth. | Able to use healthcare worker survey question data to triangulate with most subjects. |
| **Geospatial Information System Analysis** | | | | |
| Performance | This was relatively quick due to pre-existing network data. | Our approach included pre-existing road network and population density. | Collaboration with geospatial researchers using R software meant this was low cost | Validation was attempted and possible, although limited in scope. |
| Potential | Primary Geospatial Information System analysis likely to be quick, especially if prior collected data is used. | Other open-source mappings and population tools are commonly used, allowing remote analysis. | Future use of existing expertise and software can keep costs low. | Commonly overlapped with other data sources for a geospatial dimension of a subject of study. |
| **Facility Assessment** | | | | |
| Performance | Short time required. Between half and one day per facility once permission granted. | Completed with the most suitable staff member, and validated with a visual check is pragmatic. | Low cost, requiring the time of those involved only. Some individual experience required. | Able to integrate with some but not all of the barriers related to delay 3 in our matrix analysis. |
| Likely Potential | This is typical for such assessments. | The typical approach could be deepened with observing equipment in use but less practical. | Unlikely to be expensive, although permissions for studies in some facilities may attract a fee. | Measures of facility resource have been compared with other dimensions of health system access and quality. |
| **Clinical Vignettes** | | | | |
| Performance | An administered vignette taking 30 minutes per participant was fairly time-consuming. | Minimal resource requirements.  English language competence required of participants. Pragmatic purposive sampling. | Low cost related to the time of those involved. | Informed was able to evidence some but not all delay 3 barriers, in the integrated matrix analysis. |
| Potential | Smaller sampling strategies or adapting to electronic completion could speed up this approach as could multiple interviewers. | Practical sampling method could trade-off with electronic distribution. | If adapted for electronic administration, the cost could be lower still. | As a measure of care process quality, it can be validated against others such as observation, or other care quality dimensions such as structure and outcome metrics. |
