# Supplementary material for: Health system assessment for access to care after injury in low- or middle-income countries: A mixed methods study from Northern Malawi
Source: PLoS Med. 2024 Jan 22;21(1):e1004344. doi: 10.1371/journal.pmed.1004344 (PMC10843098; doi:10.1371/journal.pmed.1004344)
Supplement: Attachment — Submitted filename: Comments.docx [file pmed.1004344.s006.docx]

Dear Editor,

I have gone through this paper and thanks so much for giving me the opportunity to review this. The paper uses a Three Delays framework in order to explore the functioning of the health system in responding to trauma/injury. The researchers used a mixed methods approach.

The paper is not paged. I numbered the pages from where the title, list of authors and affiliation and this is page number 1.

Comments.

Page 6: Paragraph 2 – The authors may want to give examples of less educated health workers they are referring to.

Page 7: Paragraph 3: Only traditional leaders were invited to a meeting to be sensitized about the project. What about the rest of the community members? How did they know about this study?

Results section: In terms of barriers, the authors talk about cultural norms, religion and traditional healers. This classification is okay but religion and beliefs in traditional healers are both also cultural norms. Since they are defined later it is okay to leave them the way they are in the paper.

This paper is innovative and very well written and can be published.
