# Supplementary material for: Health system assessment for access to care after injury in low- or middle-income countries: A mixed methods study from Northern Malawi
Source: PLoS Med. 2024 Jan 22;21(1):e1004344. doi: 10.1371/journal.pmed.1004344 (PMC10843098; doi:10.1371/journal.pmed.1004344)
Supplement: Attachment — Submitted filename: Response to reviewers PLOS medicine v3.1.docx [file pmed.1004344.s007.docx]

Dear Editor

We are grateful for the opportunity to revise out manuscript. Please see below our responses to the editor and reviewer comments.

On behalf of the authors

Please respond to all editor and reviewer comments.

Please include page numbers and line numbers in the manuscript file. Use continuous line numbers (do not restart the numbering on each page). Only for review purposes, the editorial team decided to number the pages of your manuscript starting with the abstract page as 1.

We have added page and line numbers as requested.

Please cite the reference numbers in square brackets (e.g., “We used the techniques developed by our colleagues [19] to analyze the data”). Citations should be preceding punctuation.

The references were already in square brackets. We have moved citations to in front of the punctuation.

Please cite your Supporting Information as outlined here: <https://journals.plos.org/plosmedicine/s/supporting-information>.

Please ensure to be consistent in the use of numbers as words or numerals.

Please ensure to be consistent in writing ‘mixed-methods’ or ‘mixed methods’.

We have used the term ‘mixed methods’ throughout.

Please report your qualitative study according to the appropriate study design provided at <http://www.equator-network.org/?post_type=eq_guidelines&eq_guidelines_study_design=qualitative-research&eq_guidelines_clinical_specialty=0&eq_guidelines_report_section=0&s=>   and provide the relevant completed checklist. In general, we recommend that authors use the COREQ checklist, or other relevant checklists listed by the Equator Network, such as the SRQR, to ensure complete reporting (<http://journals.plos.org/plosone/s/submission-guidelines#loc-qualitative-research>). In the checklist, please include sufficient text excerpted from the manuscript to explain how you accomplished all applicable items.

This is a mixed methods study and we have used the GRAMMS principles for reporting. We consider this the most appropriate reporting guide. It is a recommended guide by [EQUATOR network.](https://www.equator-network.org/reporting-guidelines/the-quality-of-mixed-methods-studies-in-health-services-research/) We have included a GRAMMS structured checklist as supplementary material.

Please describe the conceptual framework underlying your qualitative analysis.

In general, we expect qualitative studies to include the following: 1) defined objectives or research questions; 2) description of the sampling strategy, including rationale for the recruitment method, participant inclusion/exclusion criteria and the number of participants recruited; 3) detailed reporting of the data collection procedures; 4) data analysis procedures described in sufficient detail to enable replication; 5) a discussion of potential sources of bias; and 6) a discussion of limitations.

This is a mixed methods study with a focus on reporting the novel approach and application of mixing 9 different methods, a minority (Focus group discussion, photovoice and process mapping) of which could be considered qualitative. Detail of these individual methods are included in either the referenced publication or the supplementary material.

ACADEMIC EDITOR COMMENTS

This is an innovative paper in an important area that could benefit however from the very nice set of suggestions made by reviewer 2.

Thank you. We have responded to the suggestions by reviewer 2.

COMPETING INTEREST STATEMENT

All authors must declare their relevant competing interests per the PLOS policy, which can be seen here: <https://journals.plos.org/plosmedicine/s/competing-interests>
For authors with ties to industry, please indicate whether any of the interests has a financial stake in the results of the current study.

ABSTRACT

Please ensure that all numbers presented in the abstract are present and identical to numbers presented in the main manuscript text.

We have checked and can confirm that this is the case.

PLOS Medicine requests that main results are quantified with 95% CIs as well as p values.  Please include.  When reporting p values please report as p<0.001 and where higher as the exact p value p=0.002, for example.  For the purposes of transparent data reporting, if not including the aforementioned please clearly state the reasons why not.
Please include any important dependent variables that are adjusted for in the analyses.
Throughout, suggest reporting statistical information as follows to improve clarity for the reader “22% (95% CI [13%,28%]; p</=)”. Please amend throughout the abstract and main manuscript.

Please note the use of commas to separate upper and lower bounds, as opposed to hyphens as these can be confused with reporting of negative values.
When a p value is given, please specify the statistical test used to determine it. Please report p values as p<0.001 and where higher as 'p=0.002'

In our main manuscript we do not report p values as they are not applicable. This is perhaps a generic editor comment as we don’t think it is applicable to our manuscript.

Abstract Methods and Findings:
* Please ensure that all numbers presented in the abstract are present and identical to numbers presented in the main manuscript text.

This is the case.

* Please include the study design, population and setting, number of participants, years during which the study took place, length of follow up, and main outcome measures.

We have added this where applicable.
* In the last sentence of the Abstract Methods and Findings section, please describe the main limitation(s) of the study's methodology.
limitation has been provided

“The main limitation of our study is that the framework for grading evidence strength for important health system barriers across the nine studies was done by author-derived consensus, other researchers might have created a different framework”.

Abstract Conclusions:
* Please avoid vague statements such as "these results have major implications for policy/clinical care". Mention only specific implications substantiated by the results.

We cannot find the vague statement illustrated in our manuscript.
* Please temper assertions of primacy ("Our novel approach to integrating […]”) by adding ‘to the best of our knowledge’ or similar.

We have changed this to:

“Our approach, novel to the best of our knowledge…”

p.1: Please change ‘haven’t’ to ‘have not’ and refrain from using contractions.

This has been changed
p.1: Please change ‘Delay’ to ‘delay’.

This has been changed throughout the manuscript

p.1: Please change ‘Three Delays’ to ‘three delays’.

This has been changed throughout the manuscript

p.1: Please define ‘HDSS’.
This has been defined as “Health and demographic surveillance site”

AUTHOR SUMMARY

Thank you for providing a short, non-technical Author Summary of your research to make findings accessible to a wide audience that includes both scientists and non-scientists. This text is subject to editorial change and should be distinct from the scientific abstract. The Author Summary should immediately follow the Abstract in your revised manuscript. Please see our author guidelines for more information: <https://journals.plos.org/plosmedicine/s/revising-your-manuscript#loc-author-summary>.

The summary should include 2-3 single sentence, individual bullet points under each of the questions.
It may be helpful to review currently published articles for examples which can be found on our website here <https://journals.plos.org/plosmedicine/>

This has been edited to fit the format as requested.

The author summary is pasted below:

“Why was this study done?

Injuries represent a vast and relatively neglected burden of disease affecting low- and middle-income countries (LMIC).

Whilst evidence suggests that many health systems under-perform in treating injured patients in LMICs, most assessments have not considered all elements of the healthcare system for injured people.

Innovative mixed methods approaches to holistic health system assessment including community and facility perspectives are therefore needed.

What did the researchers do and find?

Using a convergent parallel design we integrated the findings of a 1) household survey; 2) verbal autopsy analysis; 3) community focus group discussions (FGDs); 4) community photovoice; 5) facility care-pathway process mapping and elucidation of barriers following injury; 6) facility healthcare worker survey; 7) facility assessment survey; 8) clinical vignettes for care process quality assessment of facility based healthcare workers; and 9) geographic information system (GIS) analysis to examine the injury care health system in Karonga, Northern Malawi.

Using a consensus developed approach we assigned a grade related to the strength of evidence found for each method for a barrier’s importance to accessing timely quality injury care within the health system.

We identified twenty six barriers evidenced by at least three of the nine methods with the barriers “cost”, “transport” and “physical resources” having the most methods providing strong evidence they were important health system barriers within delays 1, 2 and 3 respectively.

What do these findings mean?

Integrating findings from nine diverse methods allowed a deeper understanding of the complexity of the health system for trauma care, from multiple different perpectives.

This approach allows more holisic appraisal of health system issues than single methodology studies and can prioritise barriers to address through health service development and policy.

This study, to our knowledge, represents a novel approach in both the weight and focus of a mixed-method study for health system assessment exemplifying an approach other researchers could use in similar contexts.”

INTRODUCTION

Please address past research and explain the need for and potential importance of your study. Indicate whether your study is novel and how you determined that. If there has been a systematic review of the evidence related to your study (or you have conducted one), please refer to and reference that review and indicate whether it supports the need for your study.

We have referred to a systematic review in our study justification:

“a systematic review of the literature has shown most published assessments have not considered the whole system.”

METHODS AND RESULTS

PLOS Medicine requests that main results are quantified with 95% CIs as well as p values.  Please include.  When reporting p values please report as p<0.001 and where higher as the exact p value p=0.002, for example.  For the purposes of transparent data reporting, if not including the aforementioned please clearly state the reasons why not.
Please include any important dependent variables that are adjusted for in the analyses.
Suggest reporting statistical information as detailed above – see under ABSTRACT
Please present numerators and denominators for percentages, at least in the Tables [not necessarily each time they're mentioned].

We do not report p values.

In the Methods section, be sure to refer to the Supplementary Material when introducing the different methods used to examine the health care system for injuries (Methods, Study Setting, paragraph 4).

We have moved reference to the supplementary material from paragraph 5 to paragraph 4 as suggested.

“A more detailed description of the study methods is reported in supplementary material titled unpublished methods supplementary materials.”

p.4 suggest: “Malawi is a landlocked, low-income country; the 6th poorest in the world [32]. Half of its 19 million people are children, and 50% of the population lives below the poverty line [32].”

This has been changed

p.4 suggest: “It focused specifically on the health system serving the population of Karonga HDSS in Karonga District, Northern Malawi [37].”
This has been changed

p.4 suggest: “The HDSS population is mostly rural, although about 15% live in semi-urban settlements. Half of the population lives within one kilometer of a paved road [37].”
Change made bar preserving British English spelling of kilometre

p.4. suggest: “Secondary care facilities include a government facility 70 km north and a CHAM facility 40 km south over difficult hilly terrain. Tertiary care is provided at a government facility in the regional capital, Mzuzu, 150 km south.”
This has been changed

p.5: Please change ‘Feb’ to ‘February’.
This has been changed

p.5: Please cite the specific supplementary material that you are referring to (“A more detailed description of the study methods are reported in supplementary material.”)
A more detailed description of the study methods are reported in S1 unpublished methods supplementary materials.

p.5: Please define ‘GRAMMS’.
change made “Good Reporting of A Mixed Methods Study (GRAMMS)”

p.6 suggest: “All areas of disagreement from this sample were discussed until agreement and a common understanding was reached.”
Change made

p.7: Pleasure ensure to be consistent in the use of the word ‘Delay’ (uppercade D) referring to the Three Delays or ‘delay’ in its original meaning. Please revise throughout your entire manuscript.
We have changed delay and three delays to be lower case throughout.

p.7 suggest: “Facility process mapping provided evidence for the greatest number of barriers, 25 out of 26 within the integrated analysis.”
Change made

p.7 suggest: “Six barriers within Delay 1 had strong evidence that they were important in the majority of applicable methodologies.”
Change made

p.7 suggest: “Within Delay 2, three barriers had strong evidence that they were important in the majority of applicable methods, […]”.
Change made

p.7 suggest: “For Delay 3, five barriers had strong evidence of being important in the majority of applicable methods, […]”.
Change made

DISCUSSION

Please present and organize the Discussion as follows: a short, clear summary of the article's findings; what the study adds to existing research and where and why the results may differ from previous research; strengths and limitations of the study; implications and next steps for research, clinical practice, and/or public policy; one-paragraph conclusion. Please remove any subheadings.

There are no subheadings in the discussion to remove. We have substantially edited the discussion following reflection on the suggestions made by reviewer 2 (see responses below and changes in the manuscript text).

We have added a summary paragraph which indicates next steps:

“The potential value of mixed methods in emergency health systems research is recognised, but its use is in its infancy [1]. A systematic review of the literature of trauma care system assessments in LMICs found that true mixed-methods studies integrating data before drawing conclusions are rare.[2] Our study, therefore, makes an important contribution to the existing literature in the application of mixed methods approaches to health system research in general, and trauma care system research in particular. We are not aware of any similar in-depth assessment on a single population's health system using as many as nine different methods. Our study, to our knowledge, represents a novel approach in both the weight and focus of a mixed-method study. We hope this encourages other researchers to use similar approaches to holistically assess health systems in other contexts. We believe that this approach will allow a more accurate appraisal of health system issues than other single methodology studies, influencing health service development and health system policy. Drawing on this study’s experience, funding has been granted for further holistic mixed method trauma health system assessment across four LMICs [3].”

p.11: “This is particularly the case at peripheral facilities [6, 76, 77, 80], which are routinely, as we found, the first port of call for initial management of the injured.” – Did you find this in this study? Otherwise, please add a citation or reference for the second part of this statement.
We have added “as we found in our study” to clarify.

p.13: Please temper assertions of primacy ("Our novel approach to integrating […]”) by adding ‘to the best of our knowledge’ or similar.
We have changed this:

We have changed to “Our approach, novel to the best of our knowledge,”

TABLES

Please note the use of commas to separate upper and lower bounds, as opposed to hyphens as these can be confused with reporting of negative values. Suggest reporting statistical information as detailed above – see under ABSTRACT
We have replaced hyphens with commas between numbers in tables

Please provide titles and legends for all tables (including those in Supporting Information files).
We have done this.

Please define all abbreviations used in the table below each table (including those in Supporting Information files).
We have done this

Please consider avoiding the use of red and green in order to make your figure more accessible to those with colour blindness.
Thank you, we have considered this, although on balance we feel the traffic light colour code is effective at intuitively conveying the message of the data. The overlay of evidence strength descriptors are included (silent, weak, moderate and strong) and allow the tables to be accessible and interpreted by people with colour vision deficiency. Although we would like to stick to this colouring, we would be happy to change if the editor still feels that another colour code would be more appropriate.

Table 1: Please define ‘HDSS’, ‘FGD’, ‘N/A’, ‘GIS’.
These have been defined

Table 3: Please define ‘FGD’, ‘GIS’.

These have been defined

Table 3: Please include a specific reference for “Details of these decisions are evidenced in the matrix analysis results table.”.
Change made – we have reference the appendix 1.

Table 4: Please define ‘N/A’. For clarity, please define the meaning of ‘Silent’.
Change made

Appendix 1 - Please define ‘FGD’, ‘HH’, ‘HCW’, ‘VA’, ‘D1’, ‘PV’, ‘GIS’, ‘DSS’, ‘CRH’, ‘KDH (main gov 2’)’, ‘MCH (gov 3’)’, ‘D2’, ‘WHO ETC’, ‘GP’, ‘COs', ‘MAs', ‘D3’, ‘KDH’, ‘NB’

These have been defined.

Appendix 1 – Please define ‘Silent’ as grade. We noted the use of different terms (‘Not able to assess’, ‘No evidence found’, ‘No evidence revealed’, ‘Not discussed’), please ensure to define these and if applicable, use consistent expressions.

We have edited our explanation of the term “Silent” in the methods section.

“Barriers for which a study methodology provided no evidence were categorised as silent. This silence could be a limitation of a method being unsuitable to detect evidence for a barrier, or suggestive that the barrier does not exist.”

The summary description in Appendix 1 aims to provide the justification for how a grade was reached and this will not be the same for each method or grading decision.

SUPPLEMENTARY MATERIAL

Please define all abbreviations used in the supplementary material.

p.1: Please define ‘HDSS’, ‘WHO’.
p.2: Please define ‘MEIRU’.
p.3: Please define ‘IV’.
p.4: Please define ‘CHAM’.
p.5: Please define ‘CI’.
p.6: Please define ‘VA’. Please change ‘Sep’ to ‘September’.
p.7: Please define ‘LMIC’.
p.9: Please define ‘myself’ in “non-native speakers (myself and a female Nigerian).” We suggest using initials instead.
p.13. Please change ‘72’ to ’72 hours’.
p.17: Please change ‘Jul – Oct 2019’ to ‘July – October 2019’. Please define ‘HCW’, ‘WHO ETC’.

Changes made.

Table S3: Are the numbers shown in some of the columns actual numbers from the study, or are they just examples? Please clarify.
Table S3: Please define ‘WHO’. Please change “It should take no more that 1 working day to
complete, depending on staff availability.” to “It should take no more than 1 working day to
complete, depending on staff availability.”.
Table S3: Please add headers to the different columns and explain when a field was left empty (e.g., ‘Numeric=’),
Table S3: Please define ‘HIV, ‘Hep B/C’, ‘°C’, ‘Kasi’, ‘CT’, ‘MRI’

For clarity we have relabelled table S3 as an appendix rather than a table as it is the data collection instrument used during survey data collection, rather than a table of results. We have clarified the numbers represent data entry codes. We don’t feel adding additional headers is required as it would change the structure of the tool. We have made the other changes.

REFERENCES

PLOS uses the numbered citation (citation-sequence) method and first six authors, et al.
Please ensure that journal name abbreviations match those found in the National Center for Biotechnology Information (NCBI) databases (<http://www.ncbi.nlm.nih.gov/nlmcatalog/journals>), and are appropriately formatted and capitalised.

Please also see <https://journals.plos.org/plosmedicine/s/submission-guidelines#loc-references> for further details on reference formatting.

Where website addresses are cited, please specify the date of access.

PARACHUTE RESEARCH

We note that you conducted research or obtained samples in a foreign country. Did you consider including a local author as first or last author? If not, we recommend that you consider doing so in line with ICMJE's authorship requirements (<https://www.icmje.org/recommendations/browse/roles-and-responsibilities/defining-the-role-of-authors-and-contributors.html>). PLOS has a parachute research policy which aims to promote collaboration and inclusivity in global health research. You are required to complete PLOS’ questionnaire on inclusivity in global research and submit it with your revised paper. The policy and questionnaire can be found at <https://journals.plos.org/plosone/s/best-practices-in-research-reporting>.

Some authors are from Malawi, others are not. Malawi is, therefore, a foreign country for some of the authors, but not others. Whilst we agree that parachute research should be discouraged, we also believe that first or last authorship has meaning, and giving first or last authorship to people who haven’t contributed to the extent that the meaning requires is not good practice. The authorship order reflects the contributions made in the design and conduct of the study and the writing of the manuscript. All of the authors are committed to capacity building and knowledge exchange, and although the study was primarily designed by the UK authors, authors in Malawi were given opportunities for involvement and learning at all stages of the process. We have completed an inclusivity questionnaire.

Comments from the reviewers:

Reviewer #1: I commend the authors for their novel and important work. The idea of providing multiple perspectives on each conceptual delay is useful, but having novel method to 'grade' them is an interesting approach, and offers practical value. Overall this study offers a novel and holistic approach in assessing the three delays concept in trauma system in LMICs, and this could be useful in guiding further research or aid in implementation science.

Thank you for this positive feedback.

Abstract: The abstract is satisfactory.
Introduction: This is well written and explains the context and rationale of the study.
Methods: This is well described, however having subheadings for this section will be helpful to break down the steps involved in data gathering, framework development, quantitisation, grading, scoring etc.

We have added some subheadings to break up the text including study design and mixed methods analysis.

Why were the nine methods chosen?

The methods were chosen to holistically assess the health system in its ability to provide injury care. Hence, they cover multiple aspects of care provision and access and multiple perspectives, from community to facility facing. Prior work to elucidate barriers at each of the three delay stages for injury informed discussions between authors on methodologies to assess those barriers, as have been used in this study. We have expanded the study design description accordingly.

“The study design was created following a systematic literature review on trauma health system assessment methods [2], informal literature searches on health system assessment approaches used for other health conditions [4-10], and discussion amongst co-authors. A range of methods were chosen to holistically assess the health system in its ability to provide injury care. Hence, they cover multiple aspects of care provision and access and multiple perspectives, from community to facility facing. A prior Delphi study[11] to elucidate barriers at each of the three delay stages for injury also informed discussions between authors on both which methodologies to use to assess those barriers and shaped the design of those methodologies to capture these barriers. Nine methods to examine the injury care health system were agreed upon incorporating the existing data and resources available within MEIRU. These were selected to provide multiple perspectives on each conceptual delay, encompass a range of methodological approaches, and include data from community-based and facility-based sources.”

It is also not quite clear what each method entails, perhaps adding a table to describe each method would be useful for clinicians readers to understand.

Table 1 aims to summarise the methods with more detail available in either the associated publication referenced, or the supplementary material of unpublished methods. We have added this to the table legend.

“Legend - The details of focus, purpose, sample size, and which delay they assessed are included. The table aims to summarise the methods with more detail available in either the associated publication referenced, or S1 supplementary material of unpublished method.”

Results: The results are well illustrated in the tables. The results section reads rather flat, significant results or results of importance are not highlighted or made clear. Perhaps a paragraph highlighting the barriers supported by the strongest evidence across multiple methods would be useful.

We have edited the results section to read as follows:

“The barriers, their description, and the corresponding strength of evidence that they are important within the studied health system are displayed in matrix form in summary in Table 4 and in detail in S4 Table. Within the table matrix barriers are displayed in order according to the number of methods finding strong evidence that they are important. Those barriers with the greater number of methods finding strong evidence that they were important are at the top. Within delay 1, the barrier “cost” is at the top with 5 methods finding strong and 1 method moderate evidence that it is an important health system barrier. Within delay 2 the barrier “transport” is at the top with 5 methods (all those applicable) finding strong evidence that it is an important health system barrier. Within delay 3 the barrier “physical resources” is at the top with 7 methods (all those applicable) finding strong evidence that it is an important health system barrier.

Overall, six barriers within delay 1 had strong evidence that they were important in the majority of applicable methodologies. These were “cost” (5/6), “healthcare literacy” (4/6), “cultural norms” (4/6), “traditional healers” (3/6), “perceived physical access” (3/6) and “community or bystander engagement” (3/6). Within delay 2, three barriers had strong evidence that they were important in the majority of applicable methods, *“transport*” (5/5), “*roads*” (4/5) and *“distance”* (4/5). For delay 3, five barriers had strong evidence of being important in the majority of applicable methods, *“physical resources”* (7/7), “*staff*” (6/7), “*specialists*” (5/7), “*interfacility transfer*” (5/7) and “*quality processes*” (4/7).

Facility process mapping was the method that provided evidence for the greatest number of barriers, 25 out of 26 within the integrated analysis. This was followed by the Healthcare worker survey (24 out of 26), community focus group discussions (22 out of 26) and Verbal Autopsy (18 out of 26).”

Discussion: An interpretation of why the results are noteworthy or substantial need to be highlighted. The discussion doesn't quite explain why this study is significant, and what the results add to the current literature. Overall, I believe the discussion can be made more concise in several areas.

We have substantially revised the discussion which we hope improves the focus, clarity, and implications. We have not included the adjusted text here, as this pertains to most of the discussion.

I would suggest adding the below reference in discussing aspects in the third delay, especially regarding methods in improving the quality of care. Jin et al, Effectiveness of quality improvement processes, interventions, and structure in trauma systems in low-and middle-income countries: a systematic review and meta-analysis. World journal of surgery. 2021 Jul;45(7):1982-98.

Thank you for this suggestion. We have rewritten and refocussed the discussion to emphasise the methodological learning for health system assessment so have not included this reference.

Reviewer #2: Thank you for the opportunity to review the study entitled "A holistic health system assessment for access to care after injury in low- or middle- income countries: an exemplar study from Northern Malawi." In this study, the authors implement a monumental and meticulous research methodology to analyze the trauma care system in Northern Malawi. They utilize previous literature and expert opinion to perform 9 separate studies to assess barriers to care. They identified 26 barriers present within their health system and discuss which methods were able to recognize which barriers. Overall, I found this project to be a significant contributor to the literature and believe it should be accepted after some revisions.
Thank you for this positive feedback.

My primary criticism of the paper is with the clarity in which the information is presented. This is in part related to the robustness of the study itself. While I believe this is a major strength of the project, it was difficult to keep track of what exactly was done for each of the 9 methodologies with 17 pages of supplemental material. The completeness in which this project was performed lead to an overview of several topics without proper depth of key findings. I will leave this to the publishers to decide but this project may best be presented as a series of studies in some other format rather than a single research publication.

Thank you for this reflection. We wish to emphasise the key aim of this paper was the integration of the findings from diverse methods of health system assessment to develop a rich holistic understanding of the health system. Although we agree that providing individual study result clarity, alongside the clear presentation of the mixed methods analysis is challenging, we believe that reporting each as a single study will lose the benefit of the holistic mixed methods assessment. Our substantial edits have hopefully produced better balance between the mixed-methods synthesis of the studies and the need for clarity and detail. We aim to report the results from individual methods in separate publications in time. Only a few have been published to date [12-14].

As it stands, I do believe several changes can be made.

*       It is unclear whether the primary objective of the paper is to investigate barriers to care in Karonga, Northern Malawi or develop a systematic methodology for assessing barriers in LMICs. For example, in the abstract the authors state:

"We mixed nine methods using a systems based and a three delays approach (delays in seeking, reaching, or receiving care), to achieve a deep understanding of a trauma care health system and where barrier to access to care are in Karonga, Northern Malawi as an exemplar of a low-income country health system, in addition to methods to use to maximise understanding of trauma systems."

In the introduction the authors state:

"We used a Three Delays framed holistic multi method assessment, to achieve a well-developed understanding of a trauma care health system in a low income country context [….] We also aimed to understand, if taking a frugal approach to data collection which, if any, of the methods would give the most relevant information on the state of the health system to care for injured people."

Simplifying these statements may add clarity.

We have simplified the study aims as suggested.

The abstract now reads:

“We integrated findings from nine methods using a three delays approach (delays in seeking, reaching, or receiving care) to prioritise important trauma care health system barriers in Karonga, Northern Malawi and exemplify a holistic health system assessment approach applicable in comparable settings.”

The introduction now reads:

“We used a three delays framed mixed method assessment to achieve a well-developed understanding of the whole trauma care health system in Karonga, Northern Malawi. We aimed to integrate findings from nine diverse methods to show important health system barriers to accessing quality care and thereby exemplify a holistic approach to health system assessment applicable in comparable low-income settings.”

*       There are also several confusing statements in the methods section. For example:

"Many study methods had their construction or analytical strategy informed by the Three Delays framework[49] with each considered using a framework of barriers to injury care derived from a previously published Delphi study"

Does this mean that the 9 methods selected for this study were developed with the Three Delays Model in mind? This sentence cites reference 49 while the previous citation for the methods was reference 8. Was this Delphi study where the barriers to care examples came from?

The methods were developed from the three delays concept to ensure that access to quality care for injuries was holistically understood. Hence, analysing their findings in the round (integration) informed a broad understanding of the health system for injury care.

The previous Delphi study elucidated barriers to access to quality care which are thought to occur at each of the delay stages. The authors discussed and agreed methodologies which were most likely to capture these barriers - these methodologies were selected for this study. We have edited the text and hope this is now clearer.

“Many study methods were constructed and analysed with the three delays framework specifically in mind using a framework of barriers to injury care derived from a previously published Delphi study”

We have also edited the study design section to clarify how both the systematic review and delphi study contributed to the study design.

“The study design was created following a systematic literature review on trauma health system assessment methods [2], informal literature searches on health system assessment approaches used for other health conditions [4-10], and discussion amongst co-authors. A range of methods were chosen to holistically assess the health system in its ability to provide injury care. Hence, they cover multiple aspects of care provision and access and multiple perspectives, from community to facility facing. A prior Delphi study [11] to elucidate barriers at each of the three delay stages for injury also informed discussions between authors on both which methodologies to use to assess those barriers and shaped the design of those methodologies to capture these barriers. Nine methods to examine the injury care health system were agreed upon which captured all relevant information and were feasible to deliver within MEIRU. These were selected to provide multiple perspectives on each conceptual delay, encompass a range of methodological approaches, and include data from community-based and facility-based sources”
*       I am not entirely clear about the consensus process. The methods state

"To assess this data for convergence (the extent to which findings complement or reinforce each other[20]), a consensus exercise was undertaken between three authors [….] Two authors (JW and IE) independently tested the scoring framework against a 40% sample of barriers (each of the Three Delays overall summaries and eight individual barriers) exposed by the study methods."
This could use more explanation.

We have made changes that improve clarity.

“To assess this data for convergence (the extent to which findings complement or reinforce each other [15]), a consensus exercise was undertaken between three authors (JW, IE and AD). These authors discussed a framework for how barriers identified by each method would be graded according to the evidence provided that they were an important health system barrier. For this purpose, importance of the identified health system barrier was considered a function of both a) numbers of people potentially affected and b) the amount of harm potentially caused. Using this framework agreed by these three authors, each barrier was graded as having either strong, moderate, or weak evidence that it was important within the health system. Barriers for which a study methodology provided no evidence were categorised as silent. This silence could be a limitation of a method being unsuitable to detect evidence for a barrier, or suggestive that the barrier does not exist. This framework for grading the strength of evidence from each method that a barrier is important is detailed in Table 3.”

Subsequently 2 authors tested this grading system, and once felt to be in sufficient agreement according to a weighted Cohen’s kappa, one author completed the exercise.

“Two authors (JW and IE) subsequently independently tested the agreed scoring framework against 40% sample of barriers (each of the three delays overall summaries and eight individual barriers) exposed by the study methods. A weighted Cohen’s kappa test for agreement was performed between the two authors’ gradings [16]. All areas of disagreement from this sample were discussed until agreement and a common understanding was reached. One author (JW) then continued to grade the remaining study evidence within the matrix.

.”

*       The discussion could be better organized to emphasize key points. Relating to the initial comment regarding the primary purpose of the study, the discussion explores both individual barriers as well as the convergent/divergent nature of different methods. If the primary purpose is to investigate specific barriers to care within Malawi, I would recommend exploring some of the 26 barriers in more depth. If the primary purpose is the development of the assessment than a conversation regarding convergence/divergence is warranted but deserves more details regarding strengths/limitations of different methods as well as their feasibility in implementation. As it stands, both of these are attempted leading to failure to fully explore either.

For example, if the desire is to review specific barriers, paragraph 9 has a good review of the lack of timely affordable emergency transport and its importance. This could be expanded to provider a more comprehensive overview of this topic.

Similarly, if the goal is to focus on the assessment itself, household surveys, community FGD, and facility process mapping were excellent tools to evaluate Delay 1 barriers. Exploring this finding and why this is would be useful.

Certain paragraphs could also be removed such as paragraph 8. The authors discuss the importance of financial barriers to care but this does not seem to be supported by their findings and is presented in an unclear manner. Consider removing or phasing in different way.

Thank you for these comments. The main contribution to the field we wish to bring with this paper is the novel (to the best of our knowledge) mixed methods integration of multiple studies to provide a holistic, multi perspective, barrier prioritising, health system assessment. We have substantially rewritten and restructured the discussion to accordingly following your suggestions. Due to the substantial changes made these have not been specifically pasted below but these changes are highlighted in the revised paper. Through refocusing the discussion we have expanded the text and references, whilst we think that this brings richness and clarity to the discussion, we would be happy to edit if the editor felt it necessary.

*       The authors do an excellent job incorporating previously literature into their study. While not necessary, additional studies that may be of interest include "Risk Factors for Delayed Presentation Among Patients with Musculoskeletal Injuries in Malawi" or work done by Dr. Sudha Jayaraman in Rwanda.

Thank you for drawing our attention to these important studies we have included references to some of them.

Reviewer #3: Alex McConnachie, Statistical Review

I have been asked to provide a review of the use of statistics in the paper by Whitaker et al, an assessment of barriers to access to care after injury in Malawi.

There is very little for me to review. The only statistic reported in the main body of the paper, is a weighted kappa statistic for the grading of strength of evidence, which appears correctly applied and adequately interpreted. Appendix 1 includes assorted descriptive statistics, which seem relevant. Otherwise, there seem to be no other statistical elements to the paper.

What the paper is trying to do seems very worthy, using a range of different methodologies to assess barriers to health care in a particular setting. However, I felt as though none of the individual studies was reported in sufficient detail to fully appreciate any of the findings. Appendix 1 attempts to report key outcomes in support of the grading of evidence, but each report only consists of a sentence or two - not enough to judge whether the assessment is appropriate.

The assessment process itself seemed odd to me. It seems to be conflating "impact" with "quality" of evidence, and labelling this as "strength of evidence of importance". The criteria specified in Table 3 seem clear, though it is hard to say whether they are truly comparable across study methods, and there is no obvious connection with the impact of the barrier (number affected, harm caused).

This is a challenge of mixed methods research and trying to integrate findings from studies from different philosophical paradigms of knowledge generation. But we believe that such integration is essential for identifying barriers to access to quality care, which occur across several health system domains. To evaluate the relative important of all the barriers captured in terms of patient clinical outcomes, we are currently conducting a study across 4 counties which combines integration of these methods with assessment of patient clinical outcomes, including quality of life and disability. The results from that ongoing study will be available in 4 years time. Until then, the results from the current study using broad methodologies to understand barriers active within a health system will give researchers and policy makers an approach to identify and prioritise barriers. We have included this as a limitation.

“Our framework for grading evidence strength for each barrier might have had different thresholds if different individuals were constructing it. Similarly, the kappa for agreement could have been stronger. However, our broad approach to devise and undertake a coherent analytical approach to meaningfully compare multiple different study methods remains transferable and we would encourage other researchers to further explore and tests frameworks for meaningful cross method integration and interpretation.”

We try to consider importance and define this construct in the methods. We do not attempt to report quality of evidence in the conventional evidence based medicine hierarchical sense (<https://www.ncbi.nlm.nih.gov/pmc/articles/PMC2364804/>).

The kappa statistic for independent ratings was quite good, at 0.69, but not particularly high, so I wonder whether having 60% of scenarios assessed by only one assessor is sufficiently robust.

We have added a comment in the limitation section to reflect this as above.

In short, the statistical parts of the paper are fine. However, reading the paper from the perspective of a medical statistician, I felt there was insufficient detail and rigour for the paper to be fully convincing. Saying that, this type of research is not something I would normally be connected with, so I may not be the best judge.

We welcome the perspective and accept our analytical approach is (to the best of our knowledge) novel, but believe it will make a substantial contribution to the health system literature. As above, we have now received funding to replicate the approach elsewhere, and to determine the relative importance of health system barriers identified in a quantifiable manner.


Reviewer #4

I have gone through this paper and thanks so much for giving me the opportunity to review this. The paper uses a Three Delays framework in order to explore the functioning of the health system in responding to trauma/injury. The researchers used a mixed methods approach.

The paper is not paged. I numbered the pages from where the title, list of authors and affiliation and this is page number 1.

Comments.

Page 6: Paragraph 2 – The authors may want to give examples of less educated health workers they are referring to.

We have provided an example of security staff.

Page 7: Paragraph 3: Only traditional leaders were invited to a meeting to be sensitized about the project. What about the rest of the community members? How did they know about this study?

The MEIRU usual approach to introducing community-based studies is via traditional heads who would then disseminate this information to their communities. Specific individuals invited to take part would go through an informed consent process detailed in the supplementary materials.

We have added a comment to reflect this line 326.

“…allowing onward dissemination to the community.”

Results section: In terms of barriers, the authors talk about cultural norms, religion and traditional healers. This classification is okay but religion and beliefs in traditional healers are both also cultural norms. Since they are defined later it is okay to leave them the way they are in the paper.

Thank you, we acknowledge they could be grouped in a larger classification but feel the additional granularity of meaning is useful in keeping these barriers separate.

This paper is innovative and very well written and can be published.

Thank you.

References

1.	Mehmood A, Rowther AA, Kobusingye O, Hyder AA. Assessment of pre-hospital emergency medical services in low-income settings using a health systems approach. International Journal of Emergency Medicine. 2018;11(1):53. doi: 10.1186/s12245-018-0207-6.

2.	Whitaker J, Donohoe N, Denning M, Poenaru D, Guadagno E, Leather AJM, et al. Assessing trauma care systems in low-income and middle-income countries: a systematic review and evidence synthesis mapping the Three Delays framework to injury health system assessments. BMJ global health. 2021;6(5):e004324. doi: 10.1136/bmjgh-2020-004324.

3.	NIHR Global Health Group on Equitable Access to Quality Health Care for Injured People in Four Low or Middle Income Countries: Equi-injury (NIHR133135). 2022.

4.	Ardrey J, Desmond N, Tolhurst R, Mortimer K. The Cooking and Pneumonia Study (CAPS) in Malawi: A Nested Pilot of Photovoice Participatory Research Methodology. PloS one. 2016;11(6):e0156500. Epub 2016/06/03. doi: 10.1371/journal.pone.0156500. PubMed PMID: 27254291; PubMed Central PMCID: PMCPMC4890783.

5.	Aaronson E, Mort E, Soghoian S. Mapping the process of emergency care at a teaching hospital in Ghana. Healthcare (Amsterdam, Netherlands). 2017;5(4):214-20. Epub 2017/01/10. doi: 10.1016/j.hjdsi.2016.12.001. PubMed PMID: 28065392.

6.	Vesel L, Manu A, Lohela TJ, Gabrysch S, Okyere E, Ten Asbroek AH, et al. Quality of newborn care: a health facility assessment in rural Ghana using survey, vignette and surveillance data. BMJ Open. 2013;3(5). Epub 2013/05/15. doi: 10.1136/bmjopen-2012-002326. PubMed PMID: 23667161; PubMed Central PMCID: PMCPMC3651975.

7.	Villar Uribe M, Alonge OO, Bishai DM, Bennett S. Can task-shifting work at scale?: Comparing clinical knowledge of non-physician clinicians to physicians in Nigeria. BMC Health Services Research. 2018;18(1):308. doi: 10.1186/s12913-018-3133-7.

8.	Chirowodza A, van Rooyen H, Joseph P, Sikotoyi S, Richter L, Coates T. USING PARTICIPATORY METHODS AND GEOGRAPHIC INFORMATION SYSTEMS (GIS) TO PREPARE FOR AN HIV COMMUNITY-BASED TRIAL IN VULINDLELA, SOUTH AFRICA (Project Accept–HPTN 043). Journal of community psychology. 2009;37(1):41-57. doi: 10.1002/jcop.20294. PubMed PMID: PMC2747527.

9.	Tanser FC, le Sueur D. The application of geographical information systems to important public health problems in Africa. International Journal of Health Geographics. 2002;1(1):4. doi: 10.1186/1476-072X-1-4.

10.	Makanga PT, Schuurman N, von Dadelszen P, Firoz T. A scoping review of geographic information systems in maternal health. International journal of gynaecology and obstetrics: the official organ of the International Federation of Gynaecology and Obstetrics. 2016;134(1):13-7. Epub 2016/04/01. doi: 10.1016/j.ijgo.2015.11.022. PubMed PMID: 27126906.

11.	Whitaker J, Nepogodiev D, Leather A, Davies J. Assessing barriers to quality trauma care in low and middle-income countries: A Delphi study. Injury. 2019. Epub 2019/12/31. doi: 10.1016/j.injury.2019.12.035. PubMed PMID: 31883865.

12.	Whitaker J, Amoah AS, Dube A, Chirwa L, Munthali B, Rickard RF, et al. Novel application of multi-facility process map analysis for rapid injury care health system assessment in Northern Malawi. BMJ Open. 2023;13(6):e070900. doi: 10.1136/bmjopen-2022-070900.

13.	Whitaker J, Chirwa L, Munthali B, Dube A, Amoah AS, Leather AJM, et al. Development and use of clinical vignettes to assess injury care quality in Northern Malawi. Injury. 2021. Epub 2021/01/26. doi: 10.1016/j.injury.2021.01.013. PubMed PMID: 33487406.

14.	Whitaker J, Brunelli G, Van Boeckel TP, Dube A, Amoah AS, Rickard RF, et al. Access to care following injury in Northern Malawi, a comparison of travel time estimates between Geographic Information System and community household reports. Injury. 2022. doi: <https://doi.org/10.1016/j.injury.2022.02.010>.

15.	Bazeley. P. Integrating Analyses in Mixed Methods Research. 55 City Road, London2018. Available from: <https://methods.sagepub.com/book/integrating-analyses-in-mixed-methods-research>.

16.	Altman DG. Practical statistics for medical research. London: Chapman and Hall; 1991 1991/10/01. 611 p.
