# Supplementary material for: Health system assessment for access to care after injury in low- or middle-income countries: A mixed methods study from Northern Malawi
Source: PLoS Med. 2024 Jan 22;21(1):e1004344. doi: 10.1371/journal.pmed.1004344 (PMC10843098; doi:10.1371/journal.pmed.1004344)
Supplement: Attachment — Submitted filename: Revisions.docx [file pmed.1004344.s008.docx]

Thank you for the opportunity to review this revised manuscript. I’d like to commend the authors for their dedication to health system evaluation and conducting an extensive study of the topic in a targeted population. They have made significant changes which have greatly improved the manuscript. I believe the manuscript is suitable for publication but can be enhanced if the authors address the following:

The authors state that the main purpose of this study is to integrate the findings from multiple methods to develop a holistic understanding of a health system that can be replicated in different settings. As such it would be helpful to provide a grading of the included methods to help future researchers understand which methods to use. For example, GIS evidence did not provide useful information and probably does not need to be included in future work given the limitations outlined in paragraph of the discussion 9. Similarly, do facility assessments and clinical vignettes add significant information that is not found elsewhere? The authors review this in paragraphs 7-11 and have a summary in paragraph 12 but could consider adding their recommendations for which methods to prioritize overall or for each level of delay. A simple table with this information would be useful. I think this would help the authors achieve their main goal which is to create a reproducible method for assessing trauma systems which future studies can build on.

What are the implications of these results? The study finds that the main barriers are cost, transportation, and physical resources which is well known. What additional information has been provided using multiple methods and how has the use of multiple methods added to evidence-based changes? Given the significant time and monetary investment in conducting a study such as this it is important to provide a reason why others should reproduce this work in similar settings. The study was conducted in 2019-2020 which means there has been amble time to act on these findings. Providing examples of changes that have been made or recommendations of what should be made in the future would strengthen the authors argument that a holistic approach using several different methods was useful. It is difficult to imagine others replicating this work without strong evidence of its superiority compared to less time consuming and cheaper options.
