# Supplementary material for: Health system assessment for access to care after injury in low- or middle-income countries: A mixed methods study from Northern Malawi
Source: PLoS Med. 2024 Jan 22;21(1):e1004344. doi: 10.1371/journal.pmed.1004344 (PMC10843098; doi:10.1371/journal.pmed.1004344)
Supplement: Attachment — Submitted filename: Response to editor and reviewer comments v2.0.docx [file pmed.1004344.s009.docx]

Requests from Editors:

ACADEMIC EDITOR COMMENTS

I concur with the reviewer who is asking for recommendations for future research of this type - specifically on which methods produced the most insight and is there a more efficient approach for future studies. Editor’s note: The Editorial team agrees with this comment and feels that the paper would benefit from further discussion of the implications of the study and on recommendations for future research.

We have addressed the reviewer requests – please see responses later in this document.

FINANCIAL DISCLOSURE

Please revise your Financial Disclosure statement. The funding statement should include: specific grant numbers, initials of authors who received each award, URLs to sponsors’ websites.

Specific grant numbers were not provided. All funding was awarded to JW. We have edited the financial disclosure statement as follows:

JW was awarded funding from the Drummond Committee of the Royal Army Medical Corps Charity (https://www.ramcassociation.org.uk/about/the-role-of-the-rhq-ramc), the Royal College of Surgeons of England research fellowship scheme (https://www.rcseng.ac.uk/standards-and-research/research/fellowships-awards-grants/fellowships/one-year-surgical-research-fellowship/) and the King’s centre for global health partnerships (https://www.kcl.ac.uk/kghp). There are no specific grant numbers associated with these awarded funds. The funders had no role in study design, data collection and analysis, decision to publish, or preparation of the manuscript.

TITLE

We suggest changing the title to “Health system assessment for access to care after injury in low- or middle-income countries: A mixed methods study from Northern Malawi.” or similar.

Title changed as suggested:

Health system assessment for access to care after injury in low- or middle-income countries: A mixed methods study from Northern Malawi

ABSTRACT

1) In the second paragraph of ‘Methods and Findings’ you discuss delay 1, 2 and 3 without previously specifying what delay 1, 2 and 3 are. We suggest adding this information, e.g. in line 73 “For each delay (delay 1 seeking care, delay 2 reaching care, or delay 3 receiving care)..” or similar.

We have changed the text to read:

We found that the barriers “cost”, “transport” and “physical resources” had the most methods providing strong evidence they were important health system barriers within delays 1 (seeking care), 2 (reaching care) and 3 (receiving care) respectively.

2) l.97: Please change ‘holisic’ to ‘holistic’.

We have made this change.

3) The Abstract conclusion section should be more specific to your study results. As written, the conclusion is very general and does not provide an interpretation of the study’s results presented in the abstract, such as the role of the barriers "cost", "transport" and "physical resources”. Please revise to include a more specific and objective interpretation of the data presented in the paper.

We have edited the abstract conclusion to be more specific:

By integrating nine different methods, including qualitative, quantitative, community-, patient- and healthcare worker-derived data sources we gained a rich insight into the functioning of this health system’s ability to provide injury care. This approach allowed more holistic appraisal of this health system’s issues by establishing convergence of evidence across the diverse methods used that the barriers of cost, transport and physical resources, were the most important health system barriers driving delays to seeking, reaching and receiving injury care respectively. This offers direction and confidence, over and above that derived from single methodology studies, for prioritising barriers to address through health service development and policy.

AUTHOR SUMMARY

1) Similar to comment #1 under Abstract, in the last bullet point of ‘What Did the Researchers Do and Find?’ it is not clear what delays 1, 2 and 3 signify. Please revise.

We have changed the paragraph to read:

- We found twenty six barriers evidenced by at least three of the nine methods with the barriers “cost”, “transport” and “physical resources” had the most strong evidence across methods that they were important barriers delaying seeking, reaching and receiving care, respectively.

2) Please revise the bullet points under the second subheading to ‘What Did the Researchers Do and Find?’, and describe the methods of the study in non-technical language.

The bullet points have been edited as follows:

**What did the researchers do and find?**

- To examine the injury care health system in Karonga, Northern Malawi, we integrated the findings from nine different methods; 1) household survey; 2) verbal autopsy analysis; 3) community focus group discussions (FGDs); 4) community photovoice; 5) facility care-pathway process mapping and elucidation of barriers following injury; 6) facility healthcare worker surveys; 7) facility assessment surveys; 8) clinical vignettes for care quality assessment of facility-based healthcare workers; and 9) geographic information system (GIS) analysis.
- We graded the strength of evidence each method provided as to whether a given barrier was important in inhibiting access to timely quality injury care.
- We found twenty-six barriers evidenced by at least three of the nine methods with the barriers “cost”, “transport” and “physical resources” having the strongest evidence that they were important barriers delaying seeking, reaching and receiving care, respectively.

3) l.139: Please change ‘holisic’ to ‘holistic’.

Edited bullet points as above.

4) Please revise the bullet points under the third subheading ‘What Do These Findings Mean?’ as they are currently too similar to the scientific abstract. Also, in the final bullet point of ‘What Do These Findings Mean?’ please describe the main limitations of the study in non-technical language.

We have edited the bullet points as follows:

**What do these findings mean?**

- By comparing the findings from the perspectives of nine different methods we were able to gain an in depth understanding of the health system for trauma care.
- This approach can allows researchers and planners to know the barriers consistently shown to be important and prioritise health service development and policy interventions accordingly.
- This study, to our knowledge, represents a novel and innovative approach in terms of both the number and types of methods mixed, serving as an example other researchers could use in similar contexts.
- The way we graded evidence strength for comparison across methods was somewhat subjective and other researchers may have made different judgements.

METHODS AND RESULTS

1) ll.214-215: Please provide reference.

We are not clear on the request as the following two sentences traverse lines 214-215 and are both referenced:

The HDSS has a population of over 40,000 (10,228 households) under surveillance since 2002 [37]. It is based in the surrounds of Chilumba, in the south of Karonga, a predominantly rural lakeshore district, typical of a Malawian subsistence economy community [37].

2) We suggest adding the definitions of the three delays in the Methods section as they are currently only mentioned in the Introduction.

We have edited the text as follows:

A prior Delphi study [30] to elucidate barriers at each of the three delay stages (delay 1 - seeking care, delay 2 - reaching a place of care, and delay 3 - receiving appropriate and quality care) for injury also informed discussions between authors on which methodologies to use to assess those barriers and shaped the design of those methodologies to capture these barriers.

3) l.343: Please define ‘CI’ at first use.

We have spelt out Confidence Interval as requested.

DISCUSSION

1) ll.461-466: The terms gender and sex are not interchangeable (as discussed in <https://www.who.int/health-topics/gender)>; please use the appropriate term and revise the paragraph.

We have revised the paragraph as follows:

All of our FGD groups were mixed sex. Such issues might be more likely to arise in single-sex discussion groups [68] as a sensitive topic that only community leaders felt empowered to raise.

2) ll.505-506: Please consider revising the wording to improve clarity.

We have revised the sentence as follows:

Participants cannot want something they don’t have prior knowledge of and are therefore unlikely to bring up such issues for discussion without specific prompting.

3) ll.544-546: Please provide reference.

We have added references to substantiate:

Various open and freely accessible data sources can be used to provide a theoretical spatial access dimension to the subject of interest [91, 92].

4) Please remove the subheading ‘Conclusion’. The conclusion paragraph should be part of the Discussion.

We have deleted the subheading as requested.

5) In the conclusion paragraph (ll.634-641), similarly to the Abstract, please be more specific to what your study has shown and potential implications. Lines 634-641 are almost a repetition of the Abstract’s conclusions and are rather general statements that can be mentioned earlier on in the discussion.

We have edited our discussion’s concluding paragraph as follows:

The potential value of mixed methods in emergency health systems research is recognised, but its use is in its infancy [58]. True mixed-methods studies integrating data before drawing conclusions in trauma care system assessments are rare [8]. Our study, therefore, makes an important contribution to the existing literature in the application of mixed methods approaches to health system research in general, and trauma care system research in particular. We are not aware of any similar in-depth assessment on a single population's health system using as many as nine different methods. Our study, to our knowledge, represents a novel approach in both the weight and focus of a mixed-method study. We found the barriers of cost, transportation and physical resources were most strongly evidenced across the methods. Whilst previous single method studies have highlighted these health system issues [8], our study has found them to be the most important barriers in the health system that we assessed and should end further speculation on their relative contribution to delays in accessing quality care. We hope this encourages other researchers to use similar approaches to holistically assess health systems in other contexts. Drawing on this study’s experience, funding has been granted for further holistic mixed method trauma health system assessment across four LMICs [116].

6) l.637: Please change ‘holisic’ to ‘holistic’.

Changed as requested

SUPPLEMENTARY MATERIAL

1) l.139: "Free text responses for other specified answers were reviewed by me." – please specify ‘me’ by indicating author initials.

We have changed this to:

one author (JW).

2) l.187: "we included all to review all VAs in which injury played a role." – please revise.

We have revised this as follows:

We included all underlying, direct and contributory external causes to review all VAs in which injury played a role.

3) l.260: The terms gender and sex are not interchangeable (as discussed in <https://www.who.int/health-topics/gender)>; please use the appropriate term and revise the entire supplementary material accordingly.

In the supplementary material we use “sex” on four occasions and “gender” on no occasions. We are content the appropriate term is used throughout.

4) p.16/17: Please format the references provided as described in the previous and current revision letter under REFERENCES.

We have revised the references accordingly.

5) Thank you for providing the Good Reporting of A Mixed Methods Study (GRAMMS) checklist. If possible, please format the text to a table and replace the line numbers with paragraph numbers per section (e.g. "Methods, paragraph 1"), since the page numbers of the final published paper may be different from the page numbers in the current manuscript.

The checklist has been updated accordingly.

6) S4 Table: We noted that some of the data is presented as percentages plus actual numbers, while others are presented only as actual numbers. We suggest revising the table and adding percentages where possible. For example, for "68/228 reported this amongst the top 3 overall barriers. 1st of 6 for delay 2, 3rd of 23 overall." a percentage detail might be helpful. Also, please define "hr/hrs" and make sure all abbreviations are defined.

We have added percentages where meaningful and hr has been expanded to hour.

REFERENCES

1) Please thoroughly revise all references and ensure that journal name abbreviations match those found in the National Center for Biotechnology Information (NCBI) databases (<http://www.ncbi.nlm.nih.gov/nlmcatalog/journals>), and are appropriately formatted and capitalised (e.g., for reference [2] The Lancet Global Health should be Lancet Glob Health).

2) Please thoroughly revise all references regarding completeness. For example, references [41] or [52] seem to be missing conventional citation details.

3) Where website addresses are cited, please specify the date of access (e.g. "[accessed 2021 Nov 22]").

We have revised the references accordingly.

SOCIAL MEDIA

To help us extend the reach of your research, please provide any X (formerly known as Twitter) handle(s) that would be appropriate to tag, including your own, your coauthors’, your institution, funder, or lab. Please respond to this email with any handles you wish to be included when we tweet this paper.

Please include the X handle: @drjackoids

Comments from Reviewers:

Reviewer #1: I commend the authors for conducting a well designed study on a pertinent topic. This study is the first to address the causes of delay in trauma care specific to Rwanda using rigorous methodology. The are no further changes recommended and it is ready for publication.

Thank you for the supportive feedback.

Reviewer #2: Thank you for the opportunity to review this revised manuscript. I'd like to commend the authors for their dedication to health system evaluation and conducting an extensive study of the topic in a targeted population. They have made significant changes which have greatly improved the manuscript. I believe the manuscript is suitable for publication but can be enhanced if the authors address the following:

The authors state that the main purpose of this study is to integrate the findings from multiple methods to develop a holistic understanding of a health system that can be replicated in different settings. As such it would be helpful to provide a grading of the included methods to help future researchers understand which methods to use. For example, GIS evidence did not provide useful information and probably does not need to be included in future work given the limitations outlined in paragraph of the discussion 9. Similarly, do facility assessments and clinical vignettes add significant information that is not found elsewhere? The authors review this in paragraphs 7-11 and have a summary in paragraph 12 but could consider adding their recommendations for which methods to prioritize overall or for each level of delay. A simple table with this information would be useful. I think this would help the authors achieve their main goal which is to create a reproducible method for assessing trauma systems which future studies can build on.

We have added the following text to address these points:

In the relatively under-resourced and under-researched field of trauma in LMICs [110], efficiency in health system assessments is needed to identify needs and monitor interventions [111, 112]. Rapid assessment principles appraise the potential value of methods’ suitability for inclusion in efficient and holistic mixed-method health system assessment [113]. These principles are cost-effectiveness, speed, use of multiple data sources and pragmatism [113]. Cost and speed are intersecting principles since additional resources may speed up assessment processes. Pragmatic metrics are achievable, not arduous to collect, readily interpretable, and widely applicable [114], providing sufficient rather than perfect information [113]. Triangulating findings from multiple data sources to evaluate convergence or divergence of findings is an inherent feature of mixed methods research [52].

Summarised in Table 5, with more detailed explanation in Table S5, we include our reflections on how the methods complied in this study perform in line with these principles and their potential for adaptation for future use to improve compliance with these principles of efficient health system assessment. Our reflections are summarised in a grading of well aligned, partly aligned or poorly aligned.

According to these reflections process mapping was the most compliant method, scoring as well-aligned with all 4 principles for both performance and potential. We would strongly advocate for its wider adoption by others for including in mixed-method health system assessment. Community FGDs similarly performed well and we would recommend to include this in assessments also. Pragmatic and cost considerations around administration resource requirements constrain our recommendations for inclusion of both household and health care worker surveys. Similarly, challenges in triangulating and integrating the findings from GIS, VA and clinical vignettes should be carefully considered before including in future mixed-method health system assessments. That being said, the specific purpose for a health system assessment should nuance the choice of methods included. For example, including photovoice, whilst perhaps not adding substantially to information about barriers to care in this study, could provide images that may be useful for advocating for intervention [115]. Similarly, if methods, such as process mapping, are planned to take place within a facility, including a facility assessment, may be justifiable at the same visit without adding substantially to the cost or time required for the study.

What are the implications of these results? The study finds that the main barriers are cost, transportation, and physical resources which is well known. What additional information has been provided using multiple methods and how has the use of multiple methods added to evidence-based changes? Given the significant time and monetary investment in conducting a study such as this it is important to provide a reason why others should reproduce this work in similar settings. The study was conducted in 2019-2020 which means there has been amble time to act on these findings. Providing examples of changes that have been made or recommendations of what should be made in the future would strengthen the authors argument that a holistic approach using several different methods was useful. It is difficult to imagine others replicating this work without strong evidence of its superiority compared to less time consuming and cheaper options.

As a response to previous reviewer comments our revised discussion has focussed primarily on the methodological learning rather than necessarily interventions for health system strengthening related to certain findings. Whilst the reviewer is correct that the barriers of cost, transportation, and physical resources are well known, as we indicate in our introduction, these are mostly known from single method study. Our study showed that these were the most important barriers when considering the whole health system and therefore should – at least in the health system that we assessed- end further speculation on their relative contribution to issues in accessing quality care. We have made this clear in the discussion, as follows:

We are not aware of any similar in-depth assessment on a single population's health system using as many as nine different methods. Our study, to our knowledge, represents a novel approach in both the weight and focus of a mixed-method study. We found the barriers of cost, transportation and physical resources were most strongly evidenced across the methods. Whilst previous single method studies have highlighted these health system issues [8], our study has found them to be the most important barriers in the health system that we assessed and should end further speculation on their relative contribution to delays in accessing quality care. We hope this encourages other researchers to use similar approaches to holistically assess health systems in other contexts. Drawing on this study’s experience, funding has been granted for further holistic mixed method trauma health system assessment across four LMICs [116].

Regarding the lapse of time since the study ended and submitting this manuscript for publication, this work was done as part of a PhD. The focus of the PhD student ( first author) was to conduct the individual methodological analyses, integrative analyses and ensure the PhD was submitted on time. After the PhD was awarded writing this mixed methods manuscript has had to be fitted in around a busy clinical training schedule. This has resulted in the manuscript being submitted after a brief pause since the study was done. Whereas, if these methods were used to health system assessment as part of policy and planning, the emphasis would be on analysis and write up (rather than PhD and clinical training). Other comments are addressed in responses above around the principles of rapid assessment.

Table 5: Summary of reflections on the compliance of study methods with the rapid assessment principles (authors’ judgement).

Legend - Performance refers to authors’ experience, and potential refers to the scope for adaptation to improve compliance with a rapid assessment principle. These represent the authors’ reflections.

Well-aligned (green), partly well aligned (yellow), or poorly aligned (red).

| Method |  | Speed | Pragmatism | Cost-effectiveness | Triangulation |
| --- | --- | --- | --- | --- | --- |
| Household Survey | Performance |  |  |  |  |
|  | Potential |  |  |  |  |
| Verbal Autopsy | Performance |  |  |  |  |
|  | Potential |  |  |  |  |
| Focus Group Discussions | Performance |  |  |  |  |
|  | Potential |  |  |  |  |
| Community Photovoice | Performance |  |  |  |  |
|  | Potential |  |  |  |  |
| Process mapping | Performance |  |  |  |  |
|  | Potential |  |  |  |  |
| HCW survey | Performance |  |  |  |  |
|  | Potential |  |  |  |  |
| GIS analysis | Performance |  |  |  |  |
|  | Potential |  |  |  |  |
| Clinical Vignettes | Performance |  |  |  |  |
|  | Potential |  |  |  |  |
| Facility Assessment | Performance |  |  |  |  |
|  | Potential |  |  |  |  |

**Table S5 Detail of reflections on the compliance of study methods with the rapid assessment principles (authors’ judgement).**

Legend - Performance refers to authors’ experience, and potential refers to the scope for adaptation to improve compliance with a rapid assessment principle. These represent the authors’ reflections.

Well-aligned (green), partly well aligned (yellow), or poorly aligned (red).

|  | **Speed** | **Pragmatism** | **Cost-effectiveness.** | **Triangulation with Multiple data sources** |
| --- | --- | --- | --- | --- |
| **Household Survey** | | | | |
| Performance | Data collection mostly complete inside two months including research assistant training and piloting. | Local language competence required by research assistants. Health and Demographic Surveillance Site used as sampling frame. | Most costly method used due to human resources requires. | Integrated effectively although more detailed barrier focussed questions could have targeted delays 2 and 3. |
| Potential | More resource could speed up substantially. | Likely to remain onerous to collect data. | Will remain resource intensive in proportion to sample size needed. | Can be a valuable source of community quantitative data for triangulation |
| **Verbal Autopsy Analysis** | | | | |
| Performance | Secondary data analysis although may take time sorting through narratives. | Valuable source of "good enough" data to interpret meaning about the available health system for community trauma-related deaths. | Secondary analysis of database was low cost, requiring researcher time only. | Analysis is of data collected for another purpose.  Identification of specific phenomena or barriers may not be possible (e.g. perceptions).  Misses non-fatal burden. |
| Potential | As above | Unlikely to be available outside of resourced and funded academic research organisation. | If available is cost-effective | Will miss much of burden and limited insights to those extractable. |
| **Community Focus Group Discussion** | | | | |
| Performance | Data collection was rapid although translation and transcription were delayed in my study. | Purposive sampling requires cultural insight. To identify recently injured community members benefited from an embedded research partner. | Relatively inexpensive to collate. Small costs for participant refreshments, a venue if necessary, staff for training, conducting FGDs and subsequent transcription. | Different FGD perspectives offered convergence of findings but also additional breadth. Integrated effectively with other studies. |
| Potential | Dedicated translation services would mitigate such delays. | Other pragmatic sampling strategies could be used instead. | Similar costs likely apply | Useful approach for confirming findings from other studies, focussed questions could compliment. |
| **Community Photovoice** | | | | |
| Performance | Data collection complete in one week with transcription taking two days dedicated work. | Ethical challenges include participant security, and taking photos of individuals. Emancipatory value contested. Some physical resource logistics related to photography equipment and images need overcoming. | Relatively inexpensive to collate. Cameras can be costly, but we used low-cost devices.  Management of lost cameras needs to be considered. | Useful for deepening understanding, may not generate as many new ideas as FGDs.  Might be focussed on where the participants can access. Use of the abstract was not as evident. |
| Likely Potential | Short data collection periods are common. More time could allow more reflection and photos. | Growth in smartphone access may ease logistics. | Low costs likely to remain achievable | Photo-elicitation of phenomena potentially deepening and enhancing exploration and understanding. |
| **Facility process mapping** | | | | |
| Performance | Each workshop took only a few hours with a similar time frame for map consolidation. | Pragmatic purposive staff sampling engaged a range of views. English language required with widespread electronic communication use enabling validation. | Minimal physical resource requirement and therefore cost. | Different healthcare worker perspectives encouraged.  This generated the greatest number of barrier ideas across all studies. |
| Likely Potential | Likely to remain a quick way to generate insight, determined by the number of workshops desired for a specific study. | Likely to remain highly practical, although validation may depend on electronic communication access. | Additional resources are unlikely to be necessary. May need translators if not in researcher's language. | Multiple perspectives are possible though caution over the role of hierarchical power dynamics in group workshop participation. |
| **Healthcare Worker survey** | | | | |
| Performance | The administered survey was relatively time-consuming. | Conducted in Chitumbuka needing fluent interviewers. Pragmatic sampling frame. Accessing staff limited to quiet facility times. | Two trained interviewers required. Use of electronic data capture minimised cost. | Additional barrier generation was minimal. But useful for quantitative value on perspectives. |
| Likely Potential | Self-administration could speed up, at likely cost of participation in number and breadth of participants. | Ability to conduct this remotely possible but challenging to capture the number and type of participants I did. | Self-administration could lower cost, but administration with interviewers likely necessary for similar breadth. | Able to use healthcare worker survey question data to triangulate with most subjects. |
| **GIS Analysis** | | | | |
| Performance | This was relatively quick due to pre-existing network data. | Our approach included pre-existing road network and population density. | Collaboration with geospatial researchers using R software meant this was low cost | Validation was attempted and possible, although limited in scope. |
| Potential | Primary GIS analysis likely to be quick, especially if prior collected data is used. | Other open-source mappings and population tools are commonly used, allowing remote analysis. | Future use of existing expertise and software can keep costs low. | Commonly overlapped with other data sources for a geospatial dimension of a subject of study. |
| **Facility Assessment** | | | | |
| Performance | Short time required. Between half and one day per facility once permission granted. | Completed with the most suitable staff member, and validated with a visual check is pragmatic. | Low cost, requiring the time of those involved only. Some individual experience required. | Able to integrate with some but not all of the barriers related to delay 3 in our matrix analysis. |
| Likely Potential | This is typical for such assessments. | The typical approach could be deepened with observing equipment in use but less practical. | Unlikely to be expensive, although permissions for studies in some facilities may attract a fee. | Measures of facility resource have been compared with other dimensions of health system access and quality. |
| **Clinical Vignettes** | | | | |
| Performance | An administered vignette taking 30 minuntes per participant was fairly time-consuming. | Minimal resource requirements.  English language competence required of participants. Pragmatic purposive sampling. | Low cost related to the time of those involved. | Informed was able to evidence some but not all delay 3 barriers, in the integrated matrix analysis. |
| Potential | Smaller sampling strategies, or adapting to electronic completion could speed up this approach as could multiple interviewers. | Practical sampling method could trade-off with electronic distribution. | If adapted for electronic administration, the cost could be lower still. | As a measure of care process quality, it can be validated against others such as observation, or other care quality dimensions such as structure and outcome metrics. |
